# Reduced relative fitness in hatchery‐origin Pink Salmon in two streams in Prince William Sound, Alaska

**DOI:** 10.1111/eva.13356

**Published:** 2022-03-15

**Authors:** Kyle R. Shedd, Emily A. Lescak, Christopher Habicht, E. Eric Knudsen, Tyler H. Dann, Heather A. Hoyt, Daniel J. Prince, William D. Templin

**Affiliations:** ^1^ Alaska Department of Fish & Game Anchorage Alaska USA; ^2^ Prince William Sound Science Center (PWSSC) Cordova Alaska USA

**Keywords:** Alaska, aquaculture, fisheries, fitness, GT‐seq, Hatchery, *Oncorhynchus*, pedigree, Pink Salmon, population genetics, relative reproductive success, straying

## Abstract

Previous studies generally report that hatchery‐origin Pacific Salmon (*Oncorhynchus* spp.) have lower relative reproductive success (RRS) than their natural‐origin counterparts. We estimated the RRS of Pink Salmon (*O*. *gorbuscha*) in Prince William Sound (PWS), Alaska using incomplete pedigrees. In contrast to other RRS studies, Pink Salmon have a short freshwater life history, freshwater habitats in PWS are largely unaltered by development, and sampling was conducted without the aid of dams or weirs resulting in incomplete sampling of spawning individuals. Pink Salmon released from large‐scale hatchery programs in PWS have interacted with wild populations for more than 15 generations. Hatchery populations were established from PWS populations but have subsequently been managed as separate broodstocks. Gene flow is primarily directional, from hatchery strays to wild populations. We used genetic‐based parentage analysis to estimate the RRS of a single generation of stray hatchery‐origin Pink Salmon in two streams, and across the odd‐ and even‐year lineages. Despite incomplete sampling, we assigned 1745 offspring to at least one parent. Reproductive success (RS), measured as sampled adult offspring that returned to their natal stream, was significantly lower for hatchery‐ vs. natural‐origin parents in both lineages, with RRS ranging from 0.03 to 0.47 for females and 0.05 to 0.86 for males. Generalized linear modeling for the even‐year lineage indicated that RRS was lower for hatchery‐origin fish, ranging from 0.42 to 0.60, after accounting for sample date (run timing), sample location within the stream, and fish length. Our results strongly suggest that hatchery‐origin strays have lower fitness in the wild. The consequences of reduced RRS on wild productivity depend on whether the mechanisms underlying reduced RRS are environmentally driven, and likely ephemeral, or genetically driven, and likely persistent across generations.

## 1 INTRODUCTION

The extent to which hatchery‐ and natural‐origin (i.e., salmon spawned in the wild that may have a mix of hatchery and wild ancestry) salmon interact, interbreed, and influence each other's fitness in natural systems is controversial (e.g., Araki & Schmid, 2010; Buhle et al., 2009; Evenson et al., 2018; Hilborn & Eggers, 2001; Koch & Narum, 2021; McGee, 2004; Naish et al., 2007; Pearsons, 2008; Smoker & Linley, 1997; Wertheimer et al., 2001, 2004). Relative reproductive success (RRS) is a widely used measure of fitness of hatchery‐origin salmon compared to natural‐origin salmon spawning in the same streams (e.g., Araki et al., 2008; Christie et al., 2014; Koch & Narum, 2021). In these studies, reproductive success (RS) is often defined as the number of adult offspring produced by an individual parent that return to the natal system, typically excluding any adult offspring that stray (donor strays; see table 1 in Knudsen et al., 2021 for definitions of straying terminology used in this paper) into unmonitored systems or those harvested in fisheries. While results vary based on species, hatchery broodstock practices, and the statistical power of study designs (e.g., Araki et al., 2008; Christie et al., 2014; Koch & Narum, 2021), the overall pattern across studies indicates reduced fitness of hatchery fish spawning in the natural environment. However, most RRS studies to date on Pacific Salmon (*Oncorhynchus* spp.) focus on species that spend over a year rearing in freshwater after hatching (but see Berejikian et al., 2009); populations spawning and rearing in human‐altered freshwater habitats; and study designs allowing for nearly complete sampling of all parents and offspring in the population, resulting in pedigrees in which both parents are known for most offspring.

**FIGURE 1:**
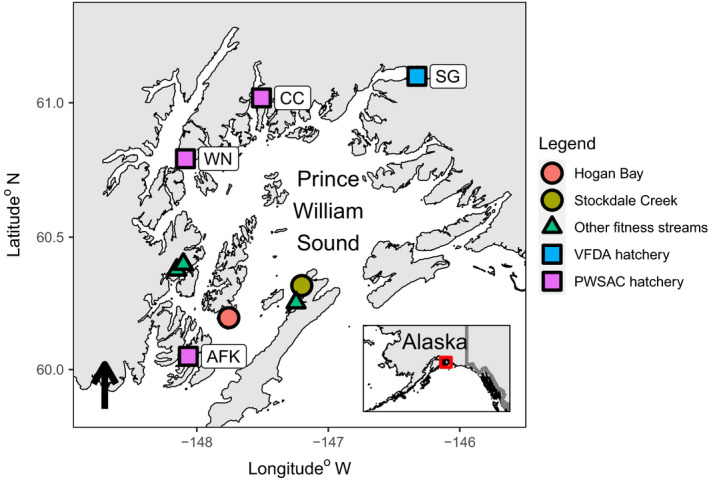
Map of Prince William Sound (PWS), Alaska, showing locations of Hogan Bay (red) and Stockdale Creek (green), additional fitness streams sampled as part of the Alaska Hatchery Research Program (AHRP) (green), and hatcheries (blue and purple). Cannery Creek (CC), Armin F. Koernig (AFK), and Wally Noerenberg (WN) are all managed by the Prince William Sound Aquaculture Corporation (PWSAC), while Solomon Gulch (SG) is managed by the Valdez Fisheries Development Association (VFDA). Inset shows the location of Prince William Sound in Alaska

In Alaska, hatcheries began practicing extensive ocean‐ranching aquaculture (i.e., fish are spawned in the hatchery, reared, and released as fry or smolts into the ocean) of Pacific Salmon in the 1970s to supplement common property fisheries and support salmon‐dependent communities. Alaskan hatcheries currently release approximately 1.8 billion juvenile salmon annually, with over 700 million Pink Salmon (*O*. *gorbuscha*) fry released in Prince William Sound (PWS; Wilson, 2020). Hatchery‐origin Pink Salmon are produced by four private nonprofit (PNP) hatcheries in PWS (Figure 1) and are differentiated from natural‐origin fish by internal thermal otolith marks applied during hatchery incubation (Volk et al., 2005). These PWS hatcheries differ from many other hatchery programs where RRS has previously been studied, not just in terms of species studied (see Koch & Narum, 2021 for a recent review), but also in sheer scale of production.

Pink Salmon have distinctive life history traits relative to other Pacific Salmon (Figure S1). First, Pink Salmon spend a shorter time (overwinter) rearing in freshwater after hatching, prior to outmigration as smolt (Groot & Margolis, 1991). Second, Pink Salmon have a fixed 2‐year generation time that results in genetically distinct odd‐ and even‐year lineages and no overlapping cohorts to buffer against interannual environmental variation. Third, Pink Salmon tend to spawn in both the freshwater and intertidal habitats of short, steep, coastal streams. The low environmental variation among spawning streams reduces the potential for local adaptations. Fourth, Pink Salmon have relatively high natural stray rates among populations, likely due to a combination of the lack of overlapping age cohorts and reduced environmental variation among spawning sites, particularly for intertidal spawners (Quinn, 2018; Salmenkova, 2017). High natural stray rates lead to increased gene flow among populations which contributes to the observed relatively low levels of genetic differentiation among stocks within lineages compared to other Pacific Salmon species (Aspinwall, 1974; Beacham et al., 2012; Cheng et al., 2016; Christensen et al., 2021; Olsen et al., 1998; Seeb et al., 1999; Tarpey et al., 2018).

Pink Salmon hatcheries in PWS were founded with broodstock from multiple donor sources local to PWS in the late 1970s and early 1980s (Habicht et al., 2000). Broodstock are currently collected at the hatcheries by volitional entry through fishways or fish ladders into brood holding ponds (PWSAC, 2021a, 2021b, 2021c; VFDA, 2021). Fish are spawned without regard to origin status, which is unknown to hatchery culturists due to the lack of external marks and inability to process hundreds of thousands of otoliths in‐season during egg take. However, otolith sampling from broodstock of three PWS Pink Salmon hatcheries in 2008 indicated that almost all broodstock (>99.7%) are of hatchery‐origin (Smoker, 2009), resulting in *de facto* segregated broodstock (see definition in Box 1 of Koch & Narum, 2021) with virtually no gene flow from wild to hatchery populations.

BOX 1 **Statistical power to detect a difference in relative reproductive success (RRS) with incomplete sampling**

1 Statistical power refers to the probability of detecting a difference between sampled distributions if there is truly a difference in the underlying distributions. In RRS studies, the statistical power to detect a difference in the reproductive success (RS) between groups, such as hatchery‐origin and natural‐origin, is affected by: (1) sample sizes of parents, (2) proportion of parents from each group (i.e., proportion of hatchery‐origin spawners), (3) proportions of offspring sampled, (4) stock productivity, and (5) effect size (Hinrichsen, 2003). Each of these variables can shape the sampled distributions of RS for each group and thus affect the ability to determine whether the distributions are statistically different from one another.

The underlying distribution of RS often approximates a negative binomial (Anderson et al., 2013; Christie et al., 2014). To illustrate the power relationship between the sample size of parents and the effect size (true RRS), Christie et al. (2014) used a heuristic approach to simulate distributions of RS and statistically compared them for different sample sizes of parents and RRS effect sizes. This work demonstrated that at least 400 parents (equal proportion hatchery and wild) would need to be sampled to detect RRS = 0.8 at least 80% of the time (power = 0.8), given their assumed distribution of RS and complete sampling of all offspring.

Here, we extend the simulation approach from Christie et al. (2014) to relax the assumed distribution of RS (i.e., stock productivity, mean, and variance of the negative binomial) and allow for incomplete sampling of offspring to more precisely estimate the statistical power of our RRS study in a natural Pink Salmon stream in PWS, Alaska. Statistical tests rely on comparing the absolute difference between sample distributions, not the relative difference. This means that anything that lowers the average RS of the sample population (i.e., incomplete sampling of offspring or low production) will inherently lower the statistical power to detect RRS < 1. Stock productivity for Pink Salmon can vary between odd‐ and even‐year lineages, as well as over time. In years of high production (high return per spawner), we expect that it would be easier to detect a difference in RS between hatchery‐ and natural‐origin spawners than in years of low productivity. For example, it is easier to differentiate a distribution of RS with an average of 8 offspring per parent from one with an average of 4 offspring per parent (RRS = 0.5) than a distribution of RS with an average of 3 and 1.5 offspring per parent (RRS = 0.5). Incomplete sampling of offspring does not affect the RRS between groups, as long as sampling is unbiased. However, incomplete sampling does lower the average RS of the sampled distribution and thus decreases the absolute difference in average RS between groups for a given effect size, making it more challenging to determine whether the distributions of RS are statistically different.

For our simulations, we wanted to determine the statistical power to detect an RRS of 0.5, the level of RRS the study was designed to detect, for a given number of hatchery‐ and natural‐origin parents sampled over a range of stock productivities (mean and variance of negative binomial) and a range of proportions of offspring sampled. We varied the mean of the negative binomial RS distribution for natural origin from 0.25 to 5, the dispersion (variance) of that distribution from 1 to 10, and the sampled proportion of offspring from 0.05 to 1. To test for differences in mean fitness (RS), we used a nonparametric permutation (randomization) test. For each combination of negative binomial mean and dispersion and offspring sampling proportion, we assigned offspring to hatchery‐ and natural‐origin parents assuming perfect genetic assignment and used a permutation test to determine whether the mean RS of hatchery‐origin fish was different than the mean RS of natural‐origin fish (RRS = 0.5). If a parent did not have any offspring assigned to it, it had an RS value of 0 (regardless of whether we knew that the parent truly did not produce any offspring or whether its offspring were not sampled). We repeated this process 2,000 times and calculated power as the proportion of trials that had a *p*‐value ≤ 0.05 (i.e., the proportion of times the true difference in RRS was statistically detected). Values for statistical power were interpolated between points to generate a heatmap based on the mean stock productivity and the offspring (F_1_) sampling proportion.

Panels A and B show the expected statistical power for Hogan Bay brood years 2013 and 2014 prior to knowing the F_1_ sampling proportion. Each of these plots assumed that the dispersion parameter for the underlying negative binomial defining RS was 1 and that the effect size was RRS = 0.5. The number of natural‐origin parents is denoted by N, and the number of hatchery‐origin parents is denoted by H, since sampling of the parental generation had already occurred when these analyses were done (winter of 2014/2015). Statistical power increases for both increasing productivity of the stock (mean RS) and increasing proportion of F_1_ offspring sampled. The yellow stars indicate the likely stock productivity of each brood year and the sampling proportion of F_1_ offspring (sampled fish/aerial survey indices). Similar analyses were performed for Stockdale as well (data not shown). The difference in expected power for RRS = 0.5 between these streams was demonstrated in our results.

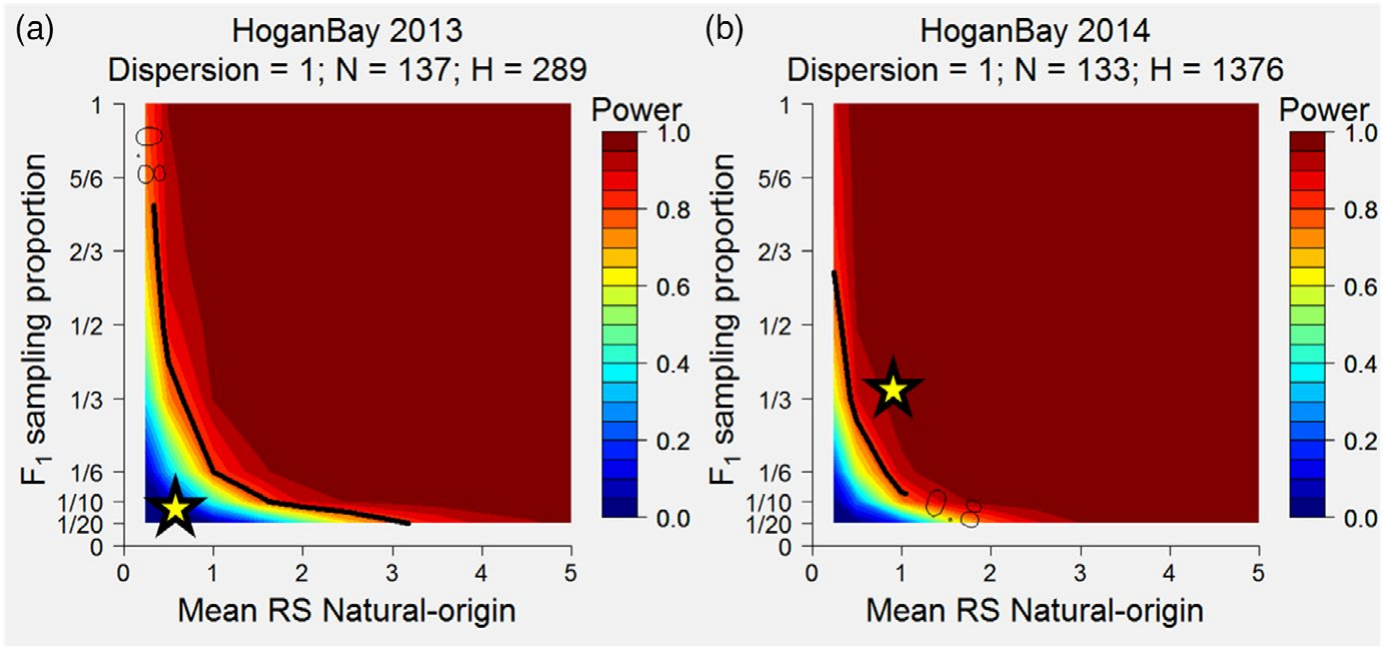

2 **ACKNOWLEDGMENTS**

Mark Christie graciously provided the R code for the simulations (Christie et al., 2014). We adapted that R code for our work here.

The commercial fishery in PWS is managed as a mixed‐stock fishery with the dual and often competing goals to both ensure wild‐stock escapement into streams and target a high proportion of hatchery‐origin Pink Salmon (Vega et al., 2019). This management strategy requires differential harvest rates of hatchery and wild stocks and is partially, but imperfectly, facilitated by spatial and temporal differences in migratory behavior between hatchery and wild fish and in‐season monitoring of hatchery proportions in the harvest via otolith sampling (Alaska Department of Fish & Game, 1994; Knudsen et al., 2021). Despite these run timing differences, there is a high degree of spatiotemporal overlap between hatchery and wild stocks in commercial fisheries occurring along migration corridors (Hilborn & Eggers, 2000; Vega et al., 2019).

The total run of hatchery‐origin Pink Salmon to PWS is much larger than that of natural‐origin Pink Salmon, with hatchery‐origin fish contributing an average of 70% of the total return of Pink Salmon to PWS between 2013 and 2015 (overlapping with the years of this study and the only three‐year period with full run reconstructions; Knudsen et al., 2021). During this period, 95%–99% of the hatchery‐origin Pink Salmon return in PWS were either harvested in common property fisheries or hatchery cost‐recovery fisheries, or taken as broodstock by the hatcheries, compared to a harvest rate of 27%–50% for natural‐origin Pink Salmon. The remaining 1%–5% of hatchery‐origin Pink Salmon that were not harvested or taken as broodstock, representing hundreds of thousands of fish annually, strayed into natural streams (donor stray rate). Hatchery‐origin spawners made up 5%–15% (recipient stray rate) of the total annual escapement for PWS Pink Salmon in 2013–2015 due to the magnitude of the total hatchery‐origin return relative to the wild return. The proportion of hatchery‐origin spawners (pHOS; recipient stray rate) in 27 sampled PWS streams ranged from 0% to 98% with higher pHOS values generally associated with smaller populations and streams located closer to hatchery release sites (Knudsen et al., 2021), as was also noted by Joyce and Evans (1999) and Brenner et al. (2012).

Concerns regarding PWS Pink Salmon hatcheries center around recipient stray rates of hatchery‐origin salmon in wild streams (pHOS), the counting of hatchery strays toward wild‐stock escapement goals, the potential for fitness declines resulting from genetic introgression, and competition between hatchery and wild stocks (Alaska Department of Fish & Game, 1994; Amoroso et al., 2017; Davis et al., 1985; Grant, 2012; Lewis et al., 2009). Some argue that hatchery‐origin Pink Salmon in PWS displace wild stocks and do not increase the net production (production after accounting for broodstock needs and hatchery cost‐recovery fisheries) above what would be expected of natural populations without hatchery supplementation (Hilborn & Eggers, 2000). Others, however, argue that hatchery‐origin fish increase harvest opportunities without negatively impacting natural stocks (Wertheimer et al., 2001, 2004). More recent analyses suggest that hatchery releases diminish the productivity of wild stocks of Pink Salmon to PWS (Amoroso et al., 2017; Ohlberger et al., 2022), despite recent record wild‐stock returns in the odd‐year lineage (Haught et al., 2017; Knudsen et al., 2021).

We hypothesized that RS differences between hatchery and wild stocks in PWS Pink Salmon due to domestication selection of hatchery fish would be smaller than what has been observed in other studies due to differences in hatchery history and practices in PWS as compared to the Pacific Northwest. These differences include (1) shorter hatchery residency (overwinter) resulting in reduced potential for domestication selection during juvenile life stages in hatcheries (Berejikian et al., 2009); (2) large hatchery broodstock sizes that reduce the likelihood of genetic divergence from wild stocks due to genetic drift, diminishing the potential for outbreeding depression when hatchery strays spawn in streams; and (3) previous and ongoing gene flow from the hatcheries to the natural populations due to hatchery straying may have already eroded local adaptations in wild stocks. We measured RRS over a single generation after 16–20 generations of hatchery production (Figure S1), in streams with consistently high pHOS and therefore high potential for previous introgression (Brenner et al., 2012; Knudsen et al., 2021). These conditions may be expected to reduce the apparent effect of hatchery‐origin on fitness, since we do not know the extent to which the natural‐origin fish in our analysis have hatchery ancestry (Willoughby & Christie, 2017).

However, many mechanisms other than domestication selection may influence RRS (reviewed by Naish et al., 2007) including: (1) relaxation of natural selection such that hatchery‐origin fish are not locally adapted to streams (Mobley et al., 2019); (2) heritable epigenetic changes due to differences between the hatchery and wild environments (Gavery et al., 2018, 2019; Le Luyer et al., 2017; Leitwein et al., 2021); (3) behavioral and ecological differences associated with broodstock sources and hatchery experience (Hughes & Murdoch, 2017; Thériault et al., 2011); and (4) study methodology (Christie et al., 2014; Hinrichsen, 2003; Koch & Narum, 2021).

The Alaska Hatchery Research Program (AHRP) was formed by the Alaska Department of Fish and Game (ADF&G) and PNP hatchery operators in 2011 to investigate these concerns by studying genetic and ecological interactions between hatchery and wild stocks in Alaska. One of the priority questions raised by the AHRP was: what is the impact of stray hatchery fish on the fitness of wild populations (Taylor, 2013)? We used genetic parentage analysis and recovery of thermally marked otoliths to estimate the RRS of hatchery‐origin Pink Salmon relative to natural‐origin Pink Salmon spawning in wild systems, as a proxy for fitness. The AHRP will eventually examine two brood years (BYs) of first‐generation RRS and one brood year (BY) of second‐generation (grandparent) effects in odd‐ and even‐year lineages of Pink Salmon for five PWS drainages (Figure S1). Here, we present results for the first generation of RRS for two PWS drainages, Hogan Bay and Stockdale creeks: short, steep, island streams that support both intertidal and upstream spawners.

This is the first study to estimate the RRS of hatchery‐origin fish in multiple remote streams without the benefit of in‐stream infrastructure to aid in sampling (i.e., dams, weirs, etc.). An underlying assumption of our study was that carcass sampling was representative of all spawners in each stream, since census sampling was logistically prohibitive. Verifying this assumption was critical, given that (1) there are known differences in run timing between wild stocks and hatchery‐origin Pink Salmon (Knudsen et al., 2021), and (2) our estimates of RRS only account for offspring that were sampled in the study streams (excluding offspring that strayed into unmonitored streams or that were harvested in commercial fisheries). Our primary objective was to test the null hypothesis that hatchery‐ and natural‐origin fish have equal RS (RRS = 1) against the alternative hypothesis that RRS is not equal to 1 by calculating unweighted RRS as the average RS of hatchery‐origin fish divided by the average RS of natural‐origin fish. Our secondary objective was to test whether run timing (sample date), spawning location (sample location), and body length differences between hatchery‐ and natural‐origin fish affected RRS by using generalized linear models (GLMs) to isolate the effect of hatchery‐origin from these potentially confounding covariates (Koch & Narum, 2021).

## 2 METHODS

### 2.1 Study sites and sampling

#### 2.1.1 Field collections

**FIGURE 2:**
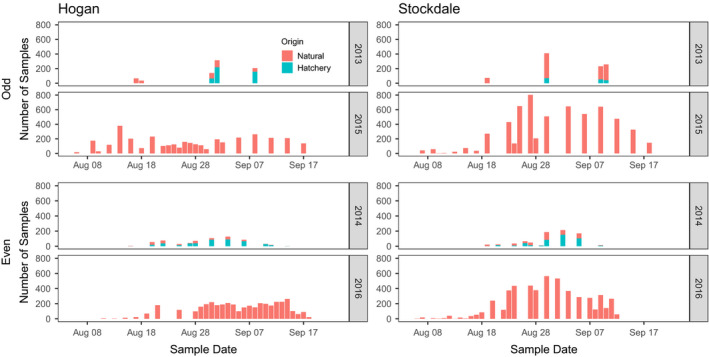
Number of Pink Salmon samples genotyped during the odd‐ and even‐year lineages from Hogan Bay and Stockdale Creek, Prince William Sound (PWS), Alaska by lineage. Within each lineage, the upper graph shows the parent year and the bottom graph shows the offspring year. Hatchery‐origin fish (strays) were excluded in the offspring year, since only natural‐origin fish could have been the offspring from the fish spawning in each creek during the parent year. Samples were collected throughout the duration of the run with sampling frequency increasing across years and higher proportions of the total escapement sampled in offspring years (2015 and 2016)

Pink Salmon were sampled in Hogan Bay Creek (60.19668°N, 147.757°W; hereafter referred to as Hogan Bay) and Stockdale Creek (60.31813°N; 147.202°W; Figure 1) from early August through late September, annually from 2013 to 2016 (Figure 2). Hogan Bay has ~550 m of stream spawning habitat, most of which is tidally influenced, whereas Stockdale has ~1500 m, much of which is above tidal influence. We relied on instream sampling from carcasses to concurrently collect genetic tissue and otolith samples after fish had the opportunity to spawn, due to the lack of external markings (i.e., adipose fin clips) to identify hatchery‐origin fish and because collecting otoliths requires destructive sampling. At times sampling was limited due to tidal stage, stream access due to flooding or high bear activity, and limited fish abundance (Knudsen et al., 2015). These limitations prevented us from collecting all potential parents and offspring in each generation and affected the statistical power of our study design (see Box 1). Global Positioning System (GPS) locations were recorded for processing areas (locations on a stream during a survey where a set of specimens were gathered, measured, and sampled), which were located at the center of, and limited to specimens collected within, a 200‐m stream reach (Knudsen et al., 2015). At each processing area, paired otolith and heart tissue samples (for DNA extraction) were collected concurrently into a 2‐mL cell of a 48 deep‐well plate and preserved in reagent alcohol (BDH1156, VWR International LLC, Radnor, PA, USA) to prevent DNA degradation (Gorman et al., 2018). Sex, length (mideye to hypural plate), and sample date were recorded for each fish. We predicted both streams were likely to have high statistical power to detect differences in RS if RRS ≤ 0.5 based on power analyses conducted after the 2014 field season (Shedd et al., 2014; Box 1). We tested for significant differences in body length, run timing (sample date), and spawning location (processing area) between hatchery‐ and natural‐origin fish using two‐sided *t*‐tests (length) or Wilcoxon tests (date and location), performed separately for each stream, sex, and lineage to determine whether these factors might explain differences in RS.

#### 2.1.2 Otolith analysis

We sent otoliths to the ADF&G Cordova Otolith Laboratory where they were analyzed for the presence of hatchery thermal marks (see Supplemental Methods).

#### 2.1.3 Genotyping

We genotyped individuals using a panel of 298 single nucleotide polymorphism (SNP) amplicons (210 single [unlinked] SNPs and 88 microhaplotypes [two linked SNPs within a single amplicon]; Table S1). We genotyped both hatchery‐ and natural‐origin fish (determined by otolith readings) for the parental run years (2013 and 2014) and only natural‐origin fish for the offspring years (2015 and 2016), as we were only interested in the returning adult offspring. The amplicons were selected specifically for parentage analysis in PWS (Dann et al. in prep) from among thousands of SNPs discovered using restriction site‐associated DNA sequencing (Baird et al., 2008) of PWS Pink Salmon collected in 2013 and 2014.

We attempted to genotype at least 500 potential parents of each origin, if available, and as many potential offspring (i.e., natural‐origin fish collected in 2015 and 2016) as possible to maximize our statistical power (Shedd et al., 2014; Box 1). We randomly subsampled individuals for genotyping within each origin from available samples with known origin based on otolith reads and known sex (see Tables 1 and S2 for sample sizes of fish genotyped and Tables S3 and S4 for sample sizes of fish collected). We followed the Genotyping‐in‐Thousands by sequencing (GT‐seq) methods described in (Campbell et al., 2015), other than deviations at the second polymerase chain reaction (PCR) step (PCR2), purification, and quantification steps (see Supplemental Methods). The final pooled library was sequenced at a concentration of 3.5 pM on an Illumina NextSeq 500 with single‐end read flow cells using 150 cycles. Postsequencing, we split reads from individual samples based on their DNA barcodes and called genotypes according to counts of amplicon‐specific alleles (Campbell et al., 2015) using GTscore (McKinney et al., 2020), modified from the default settings to reduce the maximum allowed p‐value of genotype likelihoods from 0.05 to 0.001. Genotypes were imported and archived in the ADF&G Gene Conservation Laboratory database. Genotyping quality control (QC) and quality assurance (QA) steps are described in Supplemental Methods.

**TABLE 1:** Summary of the proportion of hatchery‐origin spawners (pHOS) for the brood year (BY), numbers of individuals genotyped of hatchery‐ (H) and natural‐origin (N), offspring assigned via parentage analysis, and estimates of relative reproductive success (RRS) for both odd‐ and even‐year lineages from two streams, Hogan Bay (Hogan) and Stockdale Creek (Stockdale). Confidence intervals (CIs) for RRS were calculated following (Kalinowski & Taper, 2005)

Stream	Year	pHOS (BY)	# Genotyped								RRS (95% CI)	
			Parents		Offspring	Offspring assigned					Hatchery/natural	
			H	N		%	H	N	Dyads	Triads	Male	Female
Hogan	13/15	64%	442	321	3775	2.9%	6	104	110	0	**0.05 (0.01–0.17)**	**0.03 (0.01–0.08)**
	14/16	92%	437	214	3994	11.3%	265	208	451	22	0.86 (0.67–1.12)	**0.47 (0.37–0.62)**
Stockdale	13/15	16%	163	811	6053	2.1%	10	119	129	0	0.69 (0.31–1.35)	**0.17 (0.03–0.55)**
	14/16	74%	436	358	5199	20.3%	373	865	1055	183	**0.28 (0.24–0.34)**	**0.42 (0.35–0.50)**

Note

“Dyads” refer to single parent–offspring pairs (only one parent is known) and “Triads” refer to parent‐pair‐offspring trios (both parents are known). RRS is calculated as the average reproductive success (RS) of hatchery‐origin fish divided by the average reproductive success (RS) of natural‐origin fish. Bold RRS values indicate *p*‐value <0.05 from one sample permutation test.

### 2.2 Data analysis

#### 2.2.1 Parentage simulations

We estimated our Type I (number of individuals incorrectly assigned to parents) and Type II (number of assignments that were missed) parentage assignment error rates using simulated genotypes. We simulated 3000 offspring genotypes for each lineage from Hogan Bay using the SNP panel and assigned the simulated offspring back to parents using the pedigree reconstruction program *FRANz* (Riester et al., 2009) with the parameters in Run 1 (Table S5). We used *FRANz* because likelihood‐ and Bayesian‐based parentage analyses have been shown to perform better than exclusion‐based techniques (Anderson & Ng, 2014; Harrison et al., 2013; Hauser et al., 2011; Jones et al., 2010; Steele et al., 2013). Additionally, a full‐probability Bayesian model for pedigree reconstruction is better suited for studies that are not able to sample all potential parents and offspring because the model accounts for unsampled parents and can use sibships among sampled individuals to infer parental genotypes from offspring and fill out sparse pedigrees (Jones et al., 2010; Riester et al., 2009). Finally, we followed code from Baetscher et al. (2018) to use the CKMRsim R package (https://github.com/eriqande/CKMRsim) to evaluate the power of our SNP panel to accurately make parent–offspring and full‐ and half‐sibling assignments.

#### 2.2.2 Parentage analysis

We combined individual genotypes from our SNP panel with collection year and sex data to create input files for *FRANz*. We ran three analyses for each stream/lineage combination using the parameters in Table S5. We used genotyping error rates derived from our QC pipeline and doubled them to understand the effect of error rates on parent–offspring assignments. Values for the maximum number of potential parents by sex (N_mmax_ and N_fmax_) were based on aerial and foot survey estimates of escapement (i.e., spawning population area under the curve estimates by ADF&G that incorporated stream life and method‐specific observer efficiency; M Stopha, 2016, 2017; Vercessi, 2014, 2015). We limited the final parentage assignment to those parent–offspring pairs that had a posterior probability of assignment >90%.

#### 2.2.3 Relative reproductive success estimates

We tested the null hypothesis that RS would not differ between hatchery‐ and natural‐origin Pink Salmon by calculating RRS separately for males and females for both lineages and streams, since most of our parentage assignments were related to a single parent only (parent–offspring dyads; Table 1). These estimates based on parent–offspring dyads included all sampled potential parents (including those not assigned offspring, i.e., RS = 0). We refer to these RRS estimates as unweighted. We calculated 95% confidence intervals (CIs) around our unweighted RRS estimates following the methods of Kalinowski and Taper (2005). We tested for significant differences in RS between natural‐ and hatchery‐origin fish using a nonparametric one sample permutation test (“oneway.test” function in the coin package in R; Hothorn et al., 2006), as testing for differences in RS is equivalent to testing if RRS ≠1 (Araki & Blouin, 2005).

We tested the null hypothesis that RS would not differ among crosses between two natural‐origin parents, two hatchery‐origin parents, and one hatchery‐origin and one natural‐origin parent by calculating RS separately for the four types of crosses: hatchery–hatchery, natural–natural, hatchery–natural (hatchery female and natural male), and natural–hatchery (natural female and hatchery male). This analysis was restricted to parent‐pair‐offspring trios (triads) that produced at least one offspring (RS ≥1), as there was no way to infer that a mating occurred if RS = 0.

#### 2.2.4 Associating RS with explanatory variables

We used GLMs to test for associations between RS and parent life history variables previously shown to affect RS (Ford et al., 2012; Janowitz‐Koch et al., 2019). We restricted GLMs to streams and years with pedigrees that had at least 30 offspring assigned to each origin group. Prior to modeling, we checked for multicollinearity among variables by calculating correlation coefficients to avoid testing models containing highly correlated variables. We tested the null hypothesis that RS did not differ due to parent origin, body length, sample location (distance from stream mouth), date, or sex using a negative binomial distribution GLM with a log‐linked function (“glm.nb” function in the MASS package in R; R Core Team, 2019; Venables & Ripley, 2002). Distance from the stream mouth was determined using the R package *riverdist* (Tyers, 2020). We created the categorical variable “Intertidal” to differentiate between fish sampled in the intertidal area vs those sampled in freshwater upstream, using intertidal benchmarks derived from mean high tide coordinates provided by field crews. Sample location and intertidal were never included in the same model, as they were confounded. Following Berntson et al. (2011), we set up 131 models *a priori*, which included squared terms for body length and sample date based on visual relationships, identified statistically significant variables, and selected the best model for each stream based on Akaike's Information Criterion (AIC). We ran models with all parents combined and also separately for males and females, following Janowitz‐Koch et al. (2019). To assess model fit, we calculated the percentage of deviance explained, the GLM analog of *R* ^2^, for models from each stream and sex. The percentage of deviance explained was calculated as 1 – (residual deviance/null deviance), and is not the percentage of variance explained by the model, but rather a ratio indicating how close the model fit is to a perfect fit (interpolation) or the worst possible model (intercept only; García‐Portugués, 2021). For the top‐ranked models, we used hierarchical partitioning to determine the relative importance of each independent variable (“hier.part” function in the hier.part package in R, modified to support the negative binomial model; Chevan & Sutherland, 1991; MacNalley & Walsh, 2004).

Data and R code are available at: https://github.com/krshedd/Relative‐fitness‐of‐Pink‐Salmon, and data are openly available on the Knowledge Network for Biocomplexity (KNB) at: https://knb.ecoinformatics.org/view/doi:10.5063/F1DR2SWP.

## 3 RESULTS

### 3.1 Field collections

Field crews collected samples from a total of 46,281 individuals from Hogan Bay and Stockdale Creek, with 45,025 otoliths readable to determine the origin (Table S4). Agreement between the first and second readers was 96–97% for differentiating hatchery thermal otolith marks versus wild origins and was 93–97% for distinguishing among hatcheries for both streams combined (Jenni Morella, ADF&G Otolith Lab, pers. comm.). All PWS Pink Salmon hatcheries contributed hatchery strays during at least some of the sampling periods, with 71% overall deriving from Armin F. Koernig Hatchery (AFK), the most proximate hatchery to both streams (Figure 1; Table S4). Hatchery‐origin fish had larger average body sizes, later sample dates, and more upstream sampling locations than natural‐origin fish (Table 2).

**TABLE 2:** Body size, sample date, and distance from stream mouth for hatchery‐ and natural‐origin Pink Salmon males and females in odd‐ and even‐year lineages from two streams, Hogan Bay (Hogan) and Stockdale Creek (Stockdale)

Stream	Sex	Year	Mean length ± SD (mm)		Mean date ± SD (day)		Mean location ± SD (m)	
			Hatchery	Natural	Hatchery	Natural	Hatchery	Natural
Hogan	Male	2013	401.7 ± 24.0	398.3 ± 29.1	**Sep 3** ± **3.5**	**Aug 27** ± **7.4**	**360.5** ± **76.2**	**321.1** ± **74.4**
		2014	**435.7** ± **25.3**	**417.5** ± **31.6**	**Aug 31** ± **6.5**	**Aug 26** ± **6.2**	**353.0** ± **95.1**	**330.5** ± **85.4**
	Female	2013	**407.4** ± **20.6**	**400.6** ± **19.9**	**Sep 3** ± **3.4**	**Aug 31** ± **6.0**	369.6 ± 83.1	359.4 ± 87.7
		2014	**441.9** ± **19.4**	**434.0** ± **20.4**	**Aug 31** ± **5.5**	**Aug 29** ± **6.4**	**368.4** ± **98.7**	**340.5** ± **96.7**
Stockdale	Male	2013	390.2 ± 18.0	389.7 ± 22.9	**Sep 4** ± **5.2**	**Sep 2** ± **7.2**	**178.6** ± **218.3**	**224.4** ± **220.9**
		2014	**430.3** ± **29.1**	**419.1** ± **31.6**	**Aug 30** ± **4.1**	**Aug 26** ± **4.5**	**583.0** ± **350.8**	**395.3** ± **296.4**
	Female	2013	**398.8** ± **20.1**	**393.4** ± **19.9**	Sep 4 ± 5.3	Sep 4 ± 5.8	180.5 ± 214.6	218.1 ± 213.6
		2014	**436.6** ± **16.8**	**422.1** ± **20.3**	**Sep 1** ± **3.6**	**Aug 31** ± **4**	**668.7** ± **316.2**	**443.2** ± **304.1**

Note

Bold values indicate *p*‐value <0.05 from *t*‐test (length) or Wilcoxon test (date and location) for significance of comparison.

### 3.2 Genotyping

We selected 10,007 individuals from Hogan Bay and 15,706 individuals from Stockdale Creek for genotyping, representing an estimated 2%–54% of the escapement for a given year and stream (Tables S2 and S3). In 2015 and 2016, hatchery‐origin fish were not genotyped (Table S4). After quality assurance, we retained genotypes from 85% to 99% of Hogan Bay individuals and 74% to 93% of Stockdale Creek individuals of each origin and year for parentage analysis with a final sample size of 9183 fish from Hogan Bay and 13,020 fish from Stockdale Creek (Table S2). Variation in genotyping success tended to correlate with how degraded tissues were when sampled in the field. Final sample sizes ranged from 163 to 6053 individuals across streams, origins, and years (Tables 1 and S2). The overall background genotyping error rate among streams and years was 0.54% and ranged from 0.31 to 0.73% for Hogan Bay and 0.32%–0.71% for Stockdale Creek across years.

### 3.3 Parentage simulations

*FRANz* correctly reconstructed parent‐pair‐offspring trios for all simulated offspring from both the odd‐ and even‐year lineages, resulting in no detectable Type I or Type II error (i.e., no false or missed assignments). Simulations performed in CKMRSim demonstrated the ability of our SNP panel to distinguish between potential offspring and unrelated individuals and our known age data allowed us to unequivocally distinguish between parent–offspring and sibling relationships (Figure S3).

### 3.4 Parentage analysis

#### 3.4.1 Hogan

Exclusion probabilities from *FRANz* for our SNP panel in both the even‐ and odd‐year lineages were equal to 1.00 and all posterior probabilities of assignment were equal to 1.00. All three *FRANz* runs produced identical parentage assignments for the odd‐year lineage, while two additional offspring were assigned parents in runs 2 and 3 for the even‐year lineage (see Table S5 for run parameter values). These two individuals were excluded from downstream analyses because their posterior probabilities of assignment did not meet our cut‐off of >0.90. In the odd‐year lineage, all offspring assignments were dyads, but for the even‐year lineage, *FRANz* made 22 parent‐pair‐offspring trio assignments, which included all possible cross types (Table 1; Figure S6).

#### 3.4.2 Stockdale

In the odd‐year lineage, all cumulative exclusion probabilities were 1.00, and all posterior probabilities of assignment were equal to 1.00. All offspring assignments were dyads (Table 1). In the even‐year lineage, our sensitivity analysis in *FRANz* indicated that increasing the maximum number of parents and genotyping error rate led to one additional parent–offspring assignment, which did not meaningfully change our estimate of RRS. We report results with the more conservative escapement estimate (4038) and genotyping error rate (0.60%). The cumulative exclusion probabilities for parent assignments were all equal to 1.00 and all parentage assignments had a posterior probability of 1.00, except for four individuals whose assignments were split among multiple potential parents. *FRANz* reconstructed both dyad and triad offspring assignments (Table 1; Figure S4).

#### 3.4.3 Relative reproductive success estimates

**FIGURE 3:**
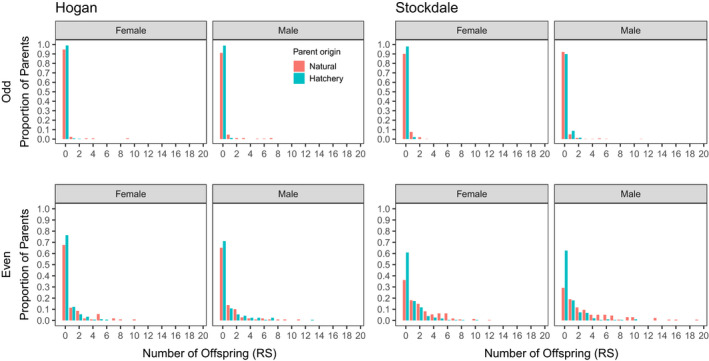
Distribution of reproductive success (RS) (number of adult offspring per parent) for female and male natural‐ and hatchery‐origin Pink Salmon for the odd (top) and even (bottom) lineages in Hogan Bay and Stockdale Creek, Prince William Sound (PWS), Alaska. Note: The x‐axis for the Hogan odd‐year lineage female plot excludes one individual assigned 41 offspring. Reproductive success was highly variable among individuals, with most potential parents assigned zero offspring and a low proportion of parents assigned high numbers of offspring

Unweighted RRS point estimates ranged from 0.03 to 0.86 and were significantly less than 1 for both streams and lineages for females, but not always significantly less than 1 for males (Table 1). Reproductive success (RS) was highly variable among individuals, varying between 0 and 41 detected offspring, with most parents assigned zero offspring (Figure 3). Offspring from all four potential types of crosses (two hatchery‐origin parents [HH], two natural‐origin parents [NN], hatchery‐origin female with natural‐origin male [HN], and natural‐origin female with hatchery‐origin male [NH]) were represented in our parent‐pair‐offspring trios for both Hogan Bay and Stockdale Creek in the even‐year lineage (Table S6; Figure S4). Reproductive success (RS) was significantly higher for crosses between two natural‐origin parents as compared to two hatchery‐origin parents for the Stockdale Creek even‐year lineage (Table S6; Figure S4). However, RS for crosses between one natural‐origin and one hatchery‐origin parent was intermediate and did not significantly differ from crosses between two hatchery‐origin or two natural‐origin parents (Table S6).

#### 3.4.4 Associating RS with explanatory variables

We used GLMs to determine the relative influence of covariates (sample date, body length, sample location, and origin) on RS for the even‐year lineage pedigrees. We did not use GLMs to test for associations between RS and parent life history variables for the odd‐year lineage due to the low number of offspring assigned to hatchery‐origin parents in both streams (Table 1).

#### 3.4.5 Hogan

**FIGURE 4:**
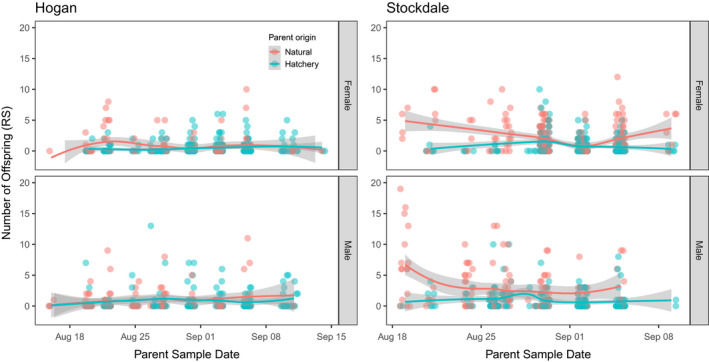
Association between reproductive success (RS) and parent sample date for Hogan Bay (Hogan) and Stockdale Creek (Stockdale) in 2014. Data for females are shown in the top plots and males in the bottom plots. Lines represent LOESS (locally weighted scatterplot smoothing) best fit with shaded areas representing 95% confidence intervals (CIs). Points are jittered on the x‐axis to prevent overplotting. Note different x‐axis scales for the two streams. While RS was variable across parent sample dates, mean RS was higher for natural‐origin fish toward the beginning and end of the run, particularly for Stockdale Creek

**FIGURE 5:**
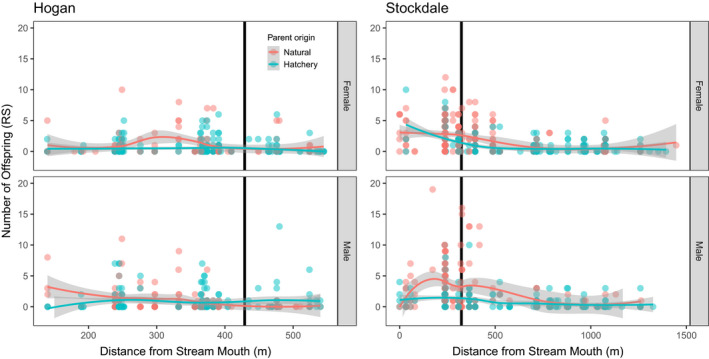
Association between reproductive success (RS) and parent sample location for Hogan Bay and Stockdale Creek in 2014. Data for females are shown in the top plots and males in the bottom plots. Lines represent LOESS (locally weighted scatterplot smoothing) best fit with shaded areas representing 95% confidence intervals (CIs). Points are jittered on the x‐axis to prevent overplotting. The vertical black line represents the upper extent of the intertidal. Note different x‐axis scales for the two streams. While RS was variable across parent sample locations, mean RS was higher near the intertidal zone

**FIGURE 6:**
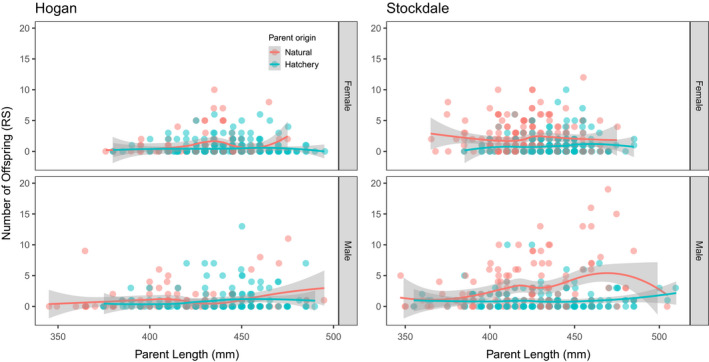
Association between reproductive success (RS) and parent length (mm) for Hogan Bay and Stockdale Creek in 2014. Data for females are shown in the top plots and males in the bottom plots. Lines represent LOESS (locally weighted scatterplot smoothing) best fit, with shaded areas representing 95% confidence intervals (CIs). Points are jittered on the x‐axis to prevent overplotting. Note different x‐axis scales for the two streams. While RS was variable across parent body length, mean RS was higher for larger natural‐origin males, particularly for Stockdale Creek

None of the explanatory variables were highly correlated (Table S7), so we included them together in the same GLMs (Table S8). The top model for females explained 6% of the deviance and included date, length, origin, and intertidal (Tables 3; S8; Figures 4, 5, 6). Origin was the most important variable in the model with 65% of the independent effects, followed by intertidal (26%), date (6%), and length (3%; Table 3). The incident ratios (exponents of model coefficients to transform out of logit link function) indicate that the modeled RRS of hatchery‐origin to natural‐origin Pink Salmon was 0.42 (95% CI: 0.24–0.71), when accounting for variation in other variables (length, intertidal, and date; Table 3). The mean number of offspring increased by ~3% for every day later that a parent was sampled and ~1% for every millimeter in parent length. Parents sampled upstream had 59% as many offspring on average as Pink Salmon sampled in the intertidal (Table 3).

**TABLE 3:** Incident ratios (95% confidence interval (CIs)), percentage of independent effects of variable contribution to the model (%IE), and overall percentage of deviance explained from top‐ranked generalized linear models (GLMs) calculated for Pink Salmon from Hogan Bay (Hogan) and Stockdale Creek (Stockdale) even‐year lineage sampled in 2014

Stream	Sex	Length (mm)	%IE	Origin	%IE	Date (day)	%IE	Distance (m)	%IE	Intertidal	%IE	% Deviance
Hogan	Females	1.01 (0.99–1.02)	3%	0.42 (0.24–0.71)	65%	1.03 (0.98–1.07)	6%	NA	NA	0.59 (0.33–1.05)	26%	6%
Hogan	Males	1.01 (1.00–1.02)	60%	NA	NA	NA	NA	0.997 (0.994–1.000)	40%	NA	NA	4%
Stockdale	Females	1.00 (1.00–1.01)	1%	0.60 (0.45–0.79)	20%	0.97 (0.94–1.00)	5%	0.998 (0.998–0.998)	74%	NA	NA	25%
Stockdale	Males	1.02 (1.01–1.02)	16%	0.43 (0.31–0.60)	28%	0.95 (0.91–0.98)	12%	0.998 (0.997–0.998)	44%	NA	NA	36%

Note

Explanatory variables include: “Length (mm)” = parent mideye to fork length, “Origin” = parent origin (hatchery‐ vs. natural‐origin), “Date (day)” = parent sample date as day of year, “Distance (m)” = parent sample location in terms of distance above the upper extent of the intertidal, and “Intertidal” = categorical variable of parent sample location within or above the upper extent of the intertidal. Incident ratios were derived from the models with the best fit, as determined by Akaike Information Criteria (AIC). Note that incident ratios are presented in the units of each variable. Variables not included in a model are indicated by NA.

The top model for males explained 4% of the deviance and included length and distance (Tables 3; S8). Length accounted for 60% of the independent effects, with the remaining 40% attributed to distance (Table 3). The mean number of offspring increased by ~1% for every millimeter increase in parent length and decreased by ~0.3% for every meter further upstream that a parent was sampled (Table 3). GLM‐derived RRS estimates were not calculated for even‐year males because origin was not a significant explanatory variable.

#### 3.4.6 Stockdale

None of the explanatory variables were highly correlated (Table S7), so we included them together in the same models. The top model for females explained 25% of the deviance and included length, distance, date, and origin (Tables 3; S9; Figures 4, 5, 6). Distance was the most important variable in the model with 74% of the independent effects, followed by origin (20%), date (5%), and length (1%; Table 3). Using the incident ratios, we calculated the modeled RRS of hatchery‐origin to natural‐origin females as 0.60 (95%CI: 0.45–0.79), when accounting for variation in other variables (length, distance, and date; Table 3). The mean number of offspring did not significantly vary with parent length, decreased by ~0.2% for every meter further upstream that a parent was sampled, and decreased by ~3% for every day later that a parent was sampled (Table 3).

The top model for males explained 36% of the deviance and included length, distance, date, and origin (Tables 3; S9; Figures 4, 5, 6). Distance was the most important variable in the model with 44% of the independent effects, followed by origin (28%), length (16%), and date (12%; Table 3). We used incident ratios from the GLMs to calculate the modeled RRS of hatchery‐origin to natural‐origin males as 0.43 (95% CI: 0.31–0.60), holding all other variables (length, distance, and date) constant. The number of offspring increased by ~2% for every millimeter increase in parent length, decreased by ~0.2% for every meter further upstream that a parent was sampled, and decreased ~5% for every day later in the season that a parent was sampled (Table 3).

## 4 DISCUSSION

This study quantified the RRS of Pink Salmon hatchery‐origin strays in PWS streams to assess the fitness impact to wild systems. Point estimates for RRS ranged from 0.03 to 0.86, which include some of the smallest RRS values ever observed in Pacific Salmon, along with estimates that are consistent with the wide ranges reported in previous studies (Christie et al., 2014). Natural‐origin parents had higher RS than hatchery‐origin parents across streams and years, although reductions in RS for male hatchery‐origin fish from Hogan Bay even‐year and Stockdale Creek odd‐year lineages were not statistically significant. However, statistical power was lower for the odd‐year lineage comparisons due to the lower number of potential parents sampled and lower offspring sampling rate (see Box 1 and Christie et al., 2014 Box 2). Additionally, we note that lineage effects (different genetic ancestries in even‐ and odd‐years) were confounded with year effects (different environmental conditions from year to year), which prevented us from disentangling the relative importance of standing genetic variation within each lineage and annual environment conditions. Ongoing work across additional years and streams within the AHRP will help to account for interannual and environmental sources of variability.

An important consideration when comparing our RRS estimates to studies in which both parents are known (parent‐pair‐offspring trios) is that our estimates of RRS are largely based on single parent assignment (parent–offspring dyads) due to incomplete sampling of potential parents. Estimates of RRS based on single parent–offspring dyad assignments would underestimate the RRS effect of hatchery‐origin fish if reductions in RS are additive. Hybrids would increase the average RS of hatchery‐origin fish (if the natural‐origin mate is unknown) and decrease the average RS of natural‐origin fish (if the hatchery‐origin mate is unknown).

Our limited cross type data suggest that crosses between two natural‐origin fish have higher RS than those between two hatchery‐origin fish, with hybrids displaying intermediate RS (Table S6). Differences in run timing between hatchery‐ and natural‐origin Pink Salmon in PWS (Knudsen et al., 2021) may reduce, but not eliminate, the potential for interbreeding. Previous genetic studies on Chum Salmon in PWS found that run timing differences between hatchery‐ and natural‐origin fish reduced, but did not completely prevent, interbreeding and introgression of hatchery alleles (Jasper et al., 2013). In steelhead, in Forks Creek on the Willapa River, Washington, interbreeding was not prevented, even though hatchery‐origin fish were selected to spawn earlier than natural‐origin individuals; up to 80% of natural‐origin steelhead were hatchery/natural hybrids (Seamons et al., 2012).

The magnitude of RRS reductions that we documented was somewhat unexpected if it is assumed that the sole mechanism for the reduction was due to domestication selection of hatchery fish. If there are heritable reductions in fitness associated with hatchery rearing, multiple generations of gene flow from hatchery‐origin individuals into wild populations might have eroded wild‐stock fitness over time. This decrease in wild‐stock fitness due to introgression would result in overestimating the relative fitness of hatchery‐origin individuals (Willoughby & Christie, 2017).

Additionally, the short hatchery residency period of Pink Salmon has been hypothesized to reduce the opportunity for domestication selection (Berejikian et al., 2009). However, modeling efforts by Baskett and Waples (2013) indicate that the timing when selection occurs is critical for predicting the fitness consequences of hatchery‐origin fish spawning in wild populations. Specifically, if natural selection occurs after reproduction and before hatchery release then hatchery‐origin fish from segregated broodstock programs may be maladapted to spawning in streams and their offspring may have lower fitness (Baskett & Waples, 2013). The low levels of genetic differentiation among PWS Pink Salmon populations and hatchery broodstocks measured at putatively neutral genetic markers (Cheng et al., 2016) do not preclude potentially important differences at adaptive loci under selection that may render hatchery strays similar enough to natural‐origin fish to survive and reproduce in streams, but different enough from natural‐origin fish to cause significant fitness declines.

Other mechanisms may also explain the observed reductions in RRS. Recent work on Steelhead and Coho Salmon has demonstrated significant epigenetic differences between hatchery and wild populations, despite nonsignificant levels of genetic differentiation (Gavery et al., 2018; Le Luyer et al., 2017). Further evidence suggests that these epigenetic differences may be heritable (Leitwein et al., 2021), despite significant within‐family effects (Gavery et al., 2019).

While every effort was made by field crews to obtain representative carcass samples, our unweighted RRS estimates were likely influenced by unrepresentative sampling by timing and/or location, given known timing differences between hatchery‐ and natural‐origin fish and potential for sampling rate differences throughout the season (Knudsen et al., 2015). Low sampling rate and high escapements in 2013 resulted in suboptimal sampling of potential parents and therefore low offspring assignment rates in 2015 (about 2.5% for both streams). Although sampling rates were higher for the smaller runs in 2014 and offspring assignment rates increased (Table 1), there is still potential for nonrepresentative sampling to affect our unweighted RRS estimates. For both parental and offspring sampling years, field crews most likely oversampled the beginning and end of the run, when there were fewer fish, relative to the middle of the run, when the abundance was much greater and sampling all available fish was impractical (Figure 2).

Reproductive success tended to be higher in natural‐origin fish from Stockdale Creek and Hogan Bay earlier in the season, likely due to a combination of reduced density on the spawning grounds and the later run timing of hatchery‐origin fish. Higher rates of commercial fishery removals later in the season likely affected the number of adult offspring that were able to return to the streams. If high heritability values underly run timing (Dickerson et al., 2005; Smoker et al., 1998), then fisheries may preferentially target the offspring of stray hatchery‐origin parents, violating the assumption of equal harvest and stray rates of natural‐ and hatchery‐origin offspring and potentially underestimating the RS of hatchery‐origin fish. Testing these hypotheses by sampling the fishery is unfortunately impractical, given that harvests range into the tens of millions.

Hatchery‐ and natural‐origin fish were distributed in different locations in the stream (particularly for Stockdale Creek), but it is unclear why hatchery‐origin fish traveled further upstream, where RS was lower (Hughes & Murdoch, 2017). They may have experienced lower RS because they were strays and were not locally adapted to the spawning habitat (Mobley et al., 2019). Alternatively, they may have traveled further upstream to less suitable spawning habitat and avoided the intertidal zone because many of the hatchery brood sources came from upstream freshwater sites and hatchery‐origin fish imprint on freshwater sources as embryos and fry in the hatcheries (Habicht et al., 2000; Mark Stopha, 2013). If upstream locations were not sampled as consistently as the intertidal, then the RS of hatchery‐origin fish may have been underestimated if their offspring inherited their proclivity to avoid spawning in the more productive intertidal zone.

Our GLM results suggest a strong negative effect of hatchery‐origin on an individual's RS after accounting for the effect of covariates (parent sampling location, sample date within the weeks‐long term of spawning, and body length), although the percentage of deviance explained by the top models ranged from 4% to 36% (Figures 4, 5, 6; Table 3). The lower percentage of deviance explained is likely due to the high inherent variability in individual RS, despite the large population‐level differences between hatchery‐origin and natural‐origin RS. The GLM results were consistent regarding the effect of fish length (i.e., large fish had higher RS than smaller fish; consistent with Dickerson et al., 2002) and sample location (i.e., fish spawning closer to the intertidal had higher RS). While sample date, our proxy for run timing, was correlated with RS, the direction and magnitude of the effect was inconsistent among streams and years. The GLM approach is not a panacea for resolving the unweighted RRS estimates because it does not provide a method to weight RRS to obtain a representative estimate. Rather, the GLM allows us to understand how other explanatory variables may influence RRS. If we did indeed oversample the tails of the run relative to the middle, we could weight our estimates of RS based on abundance within a sampling stratum, if we had reliable abundance data. Despite the limitations of both the unweighted and GLM methods, the general conclusions from these two approaches remain the same and provide context for interpretation.

## 5 CONCLUSIONS

We measured a reduction in fitness of ~50% for hatchery strays spawning in streams for the even‐year and still lower for odd‐year lineages, despite the shorter hatchery residence period of Pink Salmon, low population genetic structure in PWS Pink Salmon, previous documentation of introgression from the hatchery fish to wild populations, and incomplete sampling of spawners for our pedigrees. These results have important implications regarding the evaluation of PWS Pink Salmon hatchery programs and their unintended impacts on wild populations. However, potential management responses will depend on the causal mechanisms underlying observed RRS reductions in these two streams and the impact of 16–20 generations of potential background introgression. The causal factors are currently unclear but may involve a combination of multiple mechanisms (reviewed by Naish et al., 2007), including genetic, epigenetic, behavioral/ecological, and/or methodological. Domestication selection in PWS Pink Salmon hatcheries may result in traits that are beneficial in the hatchery environment, but maladaptive in wild streams (Christie et al., 2012). Such traits may be passed on genetically or by heritable epigenetic changes (Leitwein et al., 2021). Alternatively, hatchery‐origin fish that stray into streams may have reduced RRS due to a lack of stream‐specific local adaptations possessed by natural‐origin fish originating from that stream. Furthermore, environmental factors such as freshwater imprinting in PWS hatcheries may cause hatchery strays to ascend further upstream into less suitable spawning habitat beyond the intertidal influence. Finally, we cannot rule out the possibility that offspring of hatchery strays may be harvested at higher rates in the commercial fishery than offspring of natural‐origin fish, due to differences in run timing and fishery management.

Future results from the ongoing AHRP study, including three additional streams with more complete sampling, will allow us to better understand the variability in RRS across streams, years, and lineages. Additionally, data from a second generation (i.e., F_0_ to F_2_) from each stream may help elucidate the extent to which fitness reductions of hatchery strays are ephemeral (i.e., mostly impacting a single generation) and likely environmentally driven, or persistent across generations and likely genetically driven. Taken together, data from this and other AHRP studies will provide information for policy makers evaluating both the benefits of hatchery programs to the economic well‐being of the fishing industry and communities relying on fishing revenues, and long‐term risks to wild stocks.

## CONFLICT OF INTEREST

Research presented in this manuscript is part of the larger Alaska Hatchery Research Program (AHRP), which has received funding from the State of Alaska, private nonprofit (PNP) hatcheries, the seafood processing industry, National Oceanic and Atmospheric Administration (NOAA) 2016 Pink Salmon Disaster funds, the North Pacific Research Board, NOAA Saltonstall‐Kennedy grant program, and the Pacific Salmon Treaty Northern Endowment Fund. This program is designed to collect information to inform the Alaska Department of Fish and Game (ADF&G) policy regarding hatchery permitting and release levels. Bill Templin, the Chief Fisheries Scientist, shapes the ADF&G policy and Chris Habicht, the ADF&G Principal Geneticist, implements the genetic policy during hatchery permit reviews. Private nonprofit (PNP) hatchery operators are supported by fish taxes and must secure permits from the State of Alaska to operate hatcheries and release fish. The seafood processing industry benefits from the PNP hatchery programs that augment wild production and stabilize harvests. The State of Alaska manages fisheries with a wild‐stock priority and the seafood industry has a vested interest in sustainable fisheries management both for third‐party certification from the Marine Stewardship Council and the United Nations Responsible Fisheries Management Program and for its long‐term viability.

## Supporting information

Figure S1

Figure S2

Figure S3

Figure S4

Table S1

Table S2‐S9

## Data Availability

The data and R code that support the findings of this study are available at: https://github.com/krshedd/Relative‐fitness‐of‐Pink‐Salmon, and data are openly available on the Knowledge Network for Biocomplexity (KNB) at: https://doi.org/10.5063/F1DR2SWP.
