# Supplementary figures and images for: Reduced relative fitness in hatchery‐origin Pink Salmon in two streams in Prince William Sound, Alaska
Source: Evol Appl. 2022 Mar 15;15(3):429–46. doi: 10.1111/eva.13356 (PMC8965367; doi:10.1111/eva.13356)

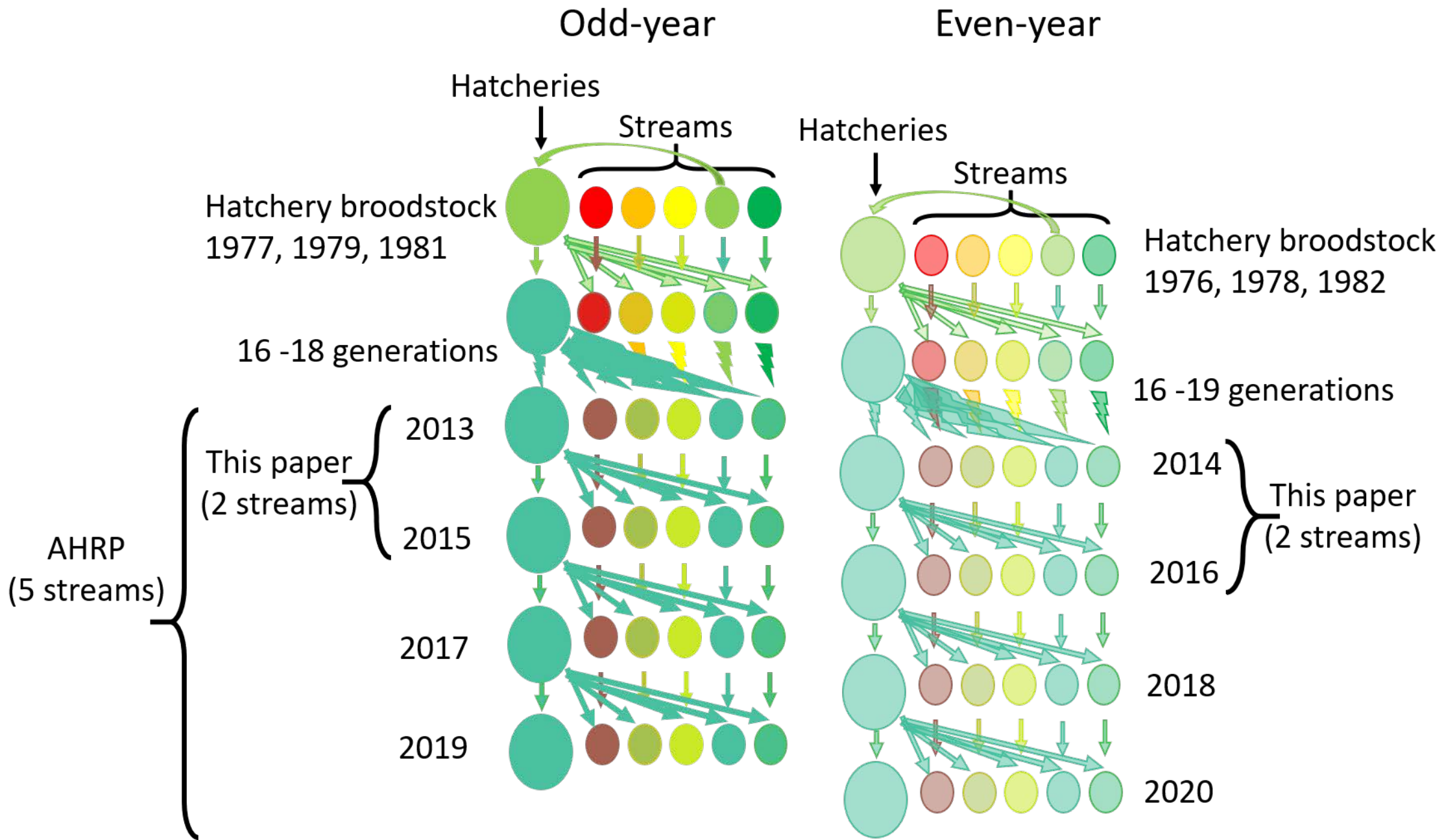

Supplement: Supplementary file 1 — Figure S1 [file EVA-15-429-s002.pdf]

Hogan

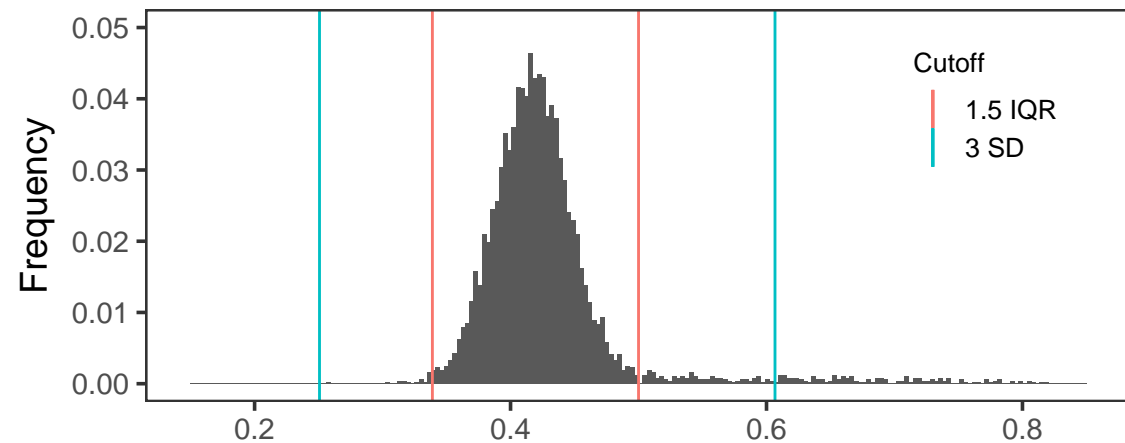

Stockdale

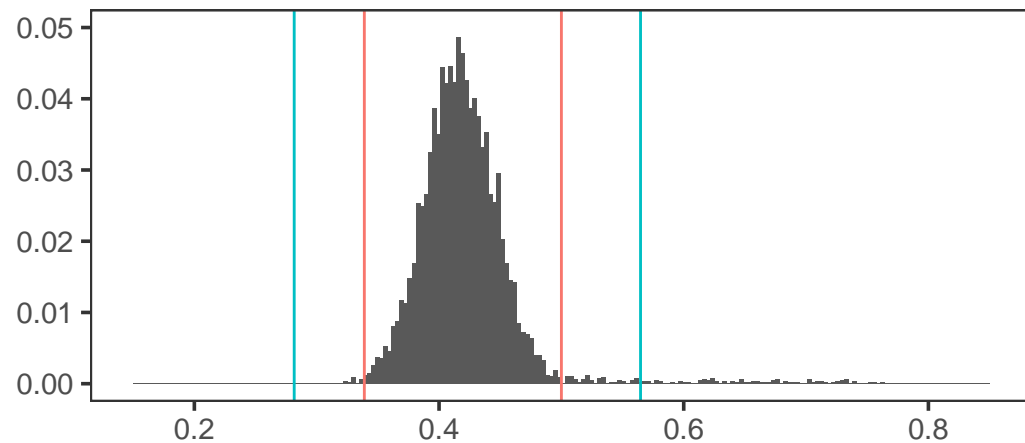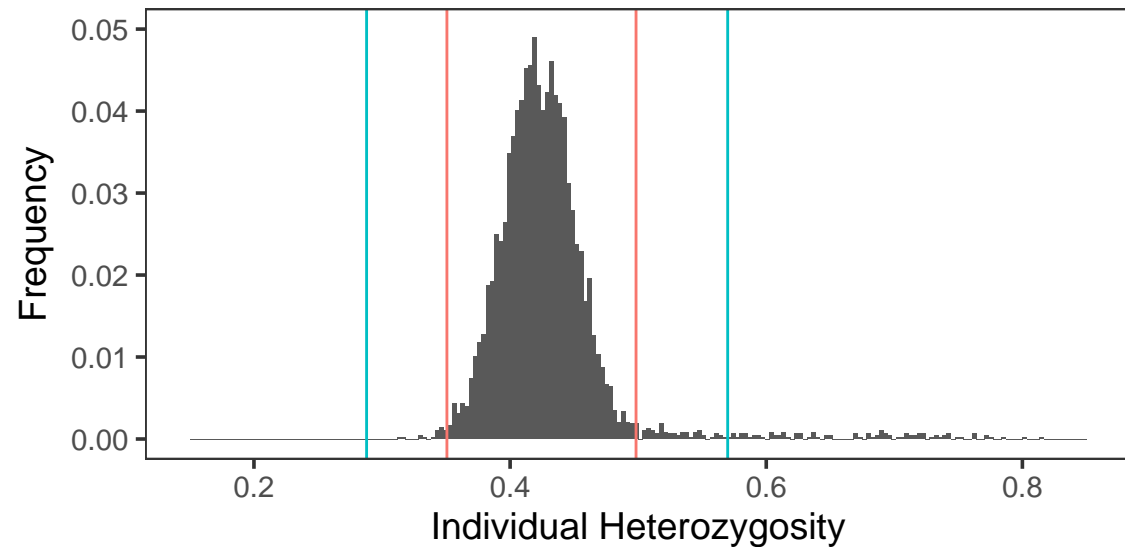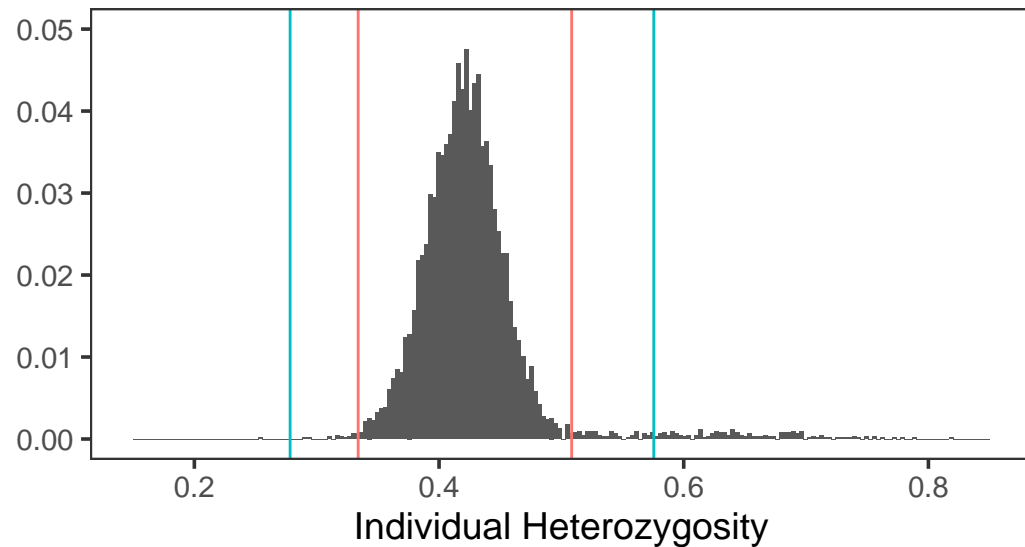

Supplement: Supplementary file 2 — Figure S2 [file EVA-15-429-s004.pdf]

Hogan

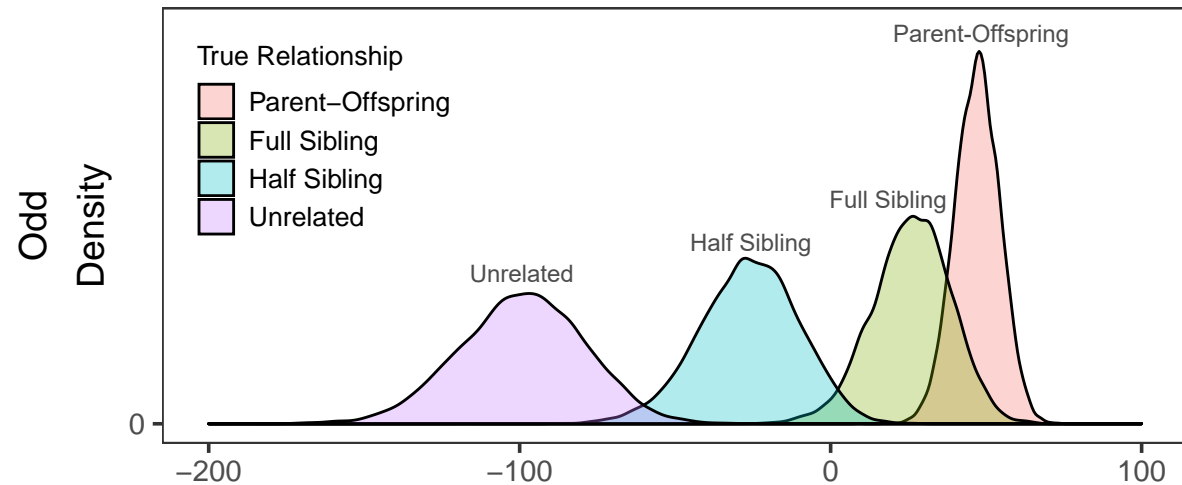

Stockdale

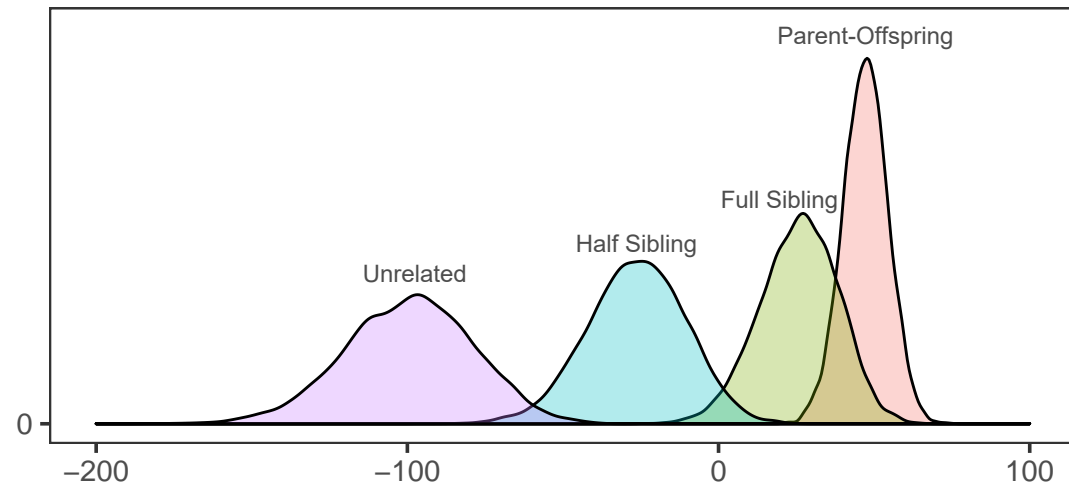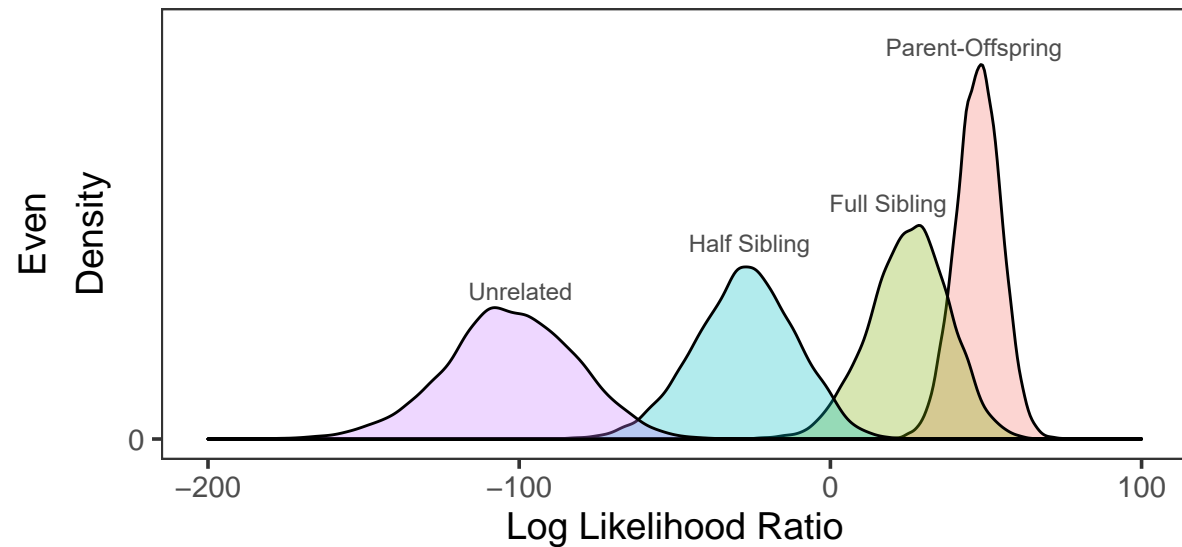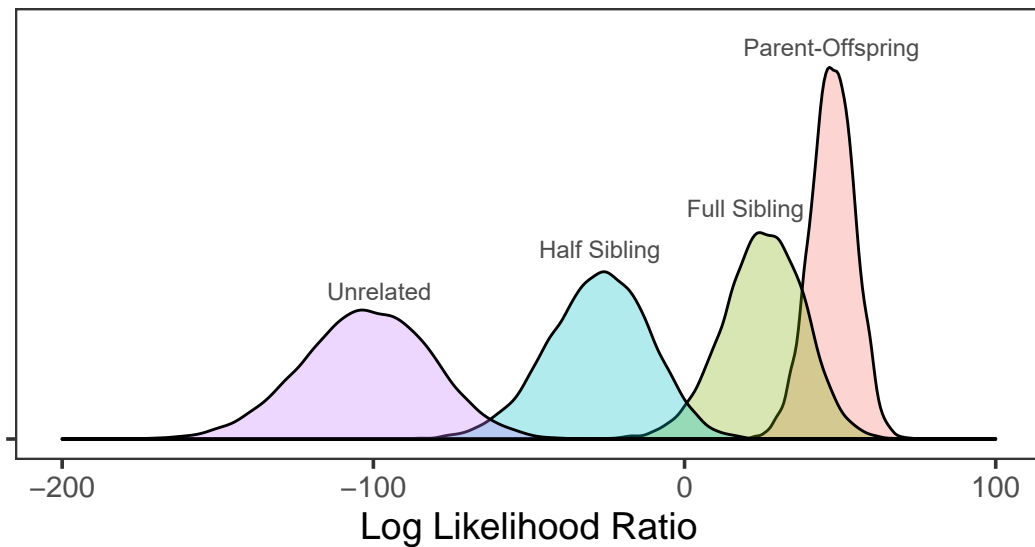

Supplement: Supplementary file 3 — Figure S3 [file EVA-15-429-s003.pdf]

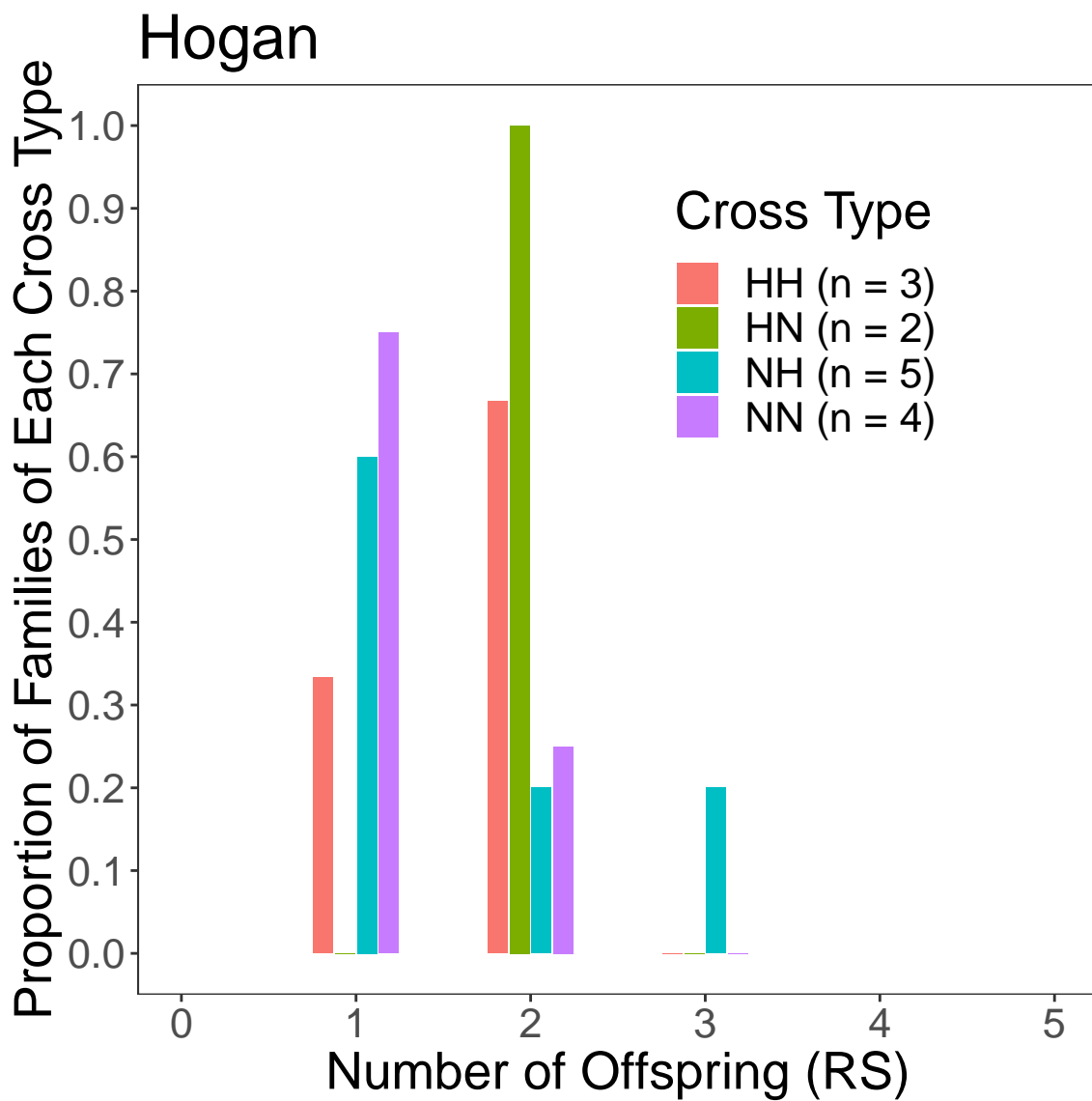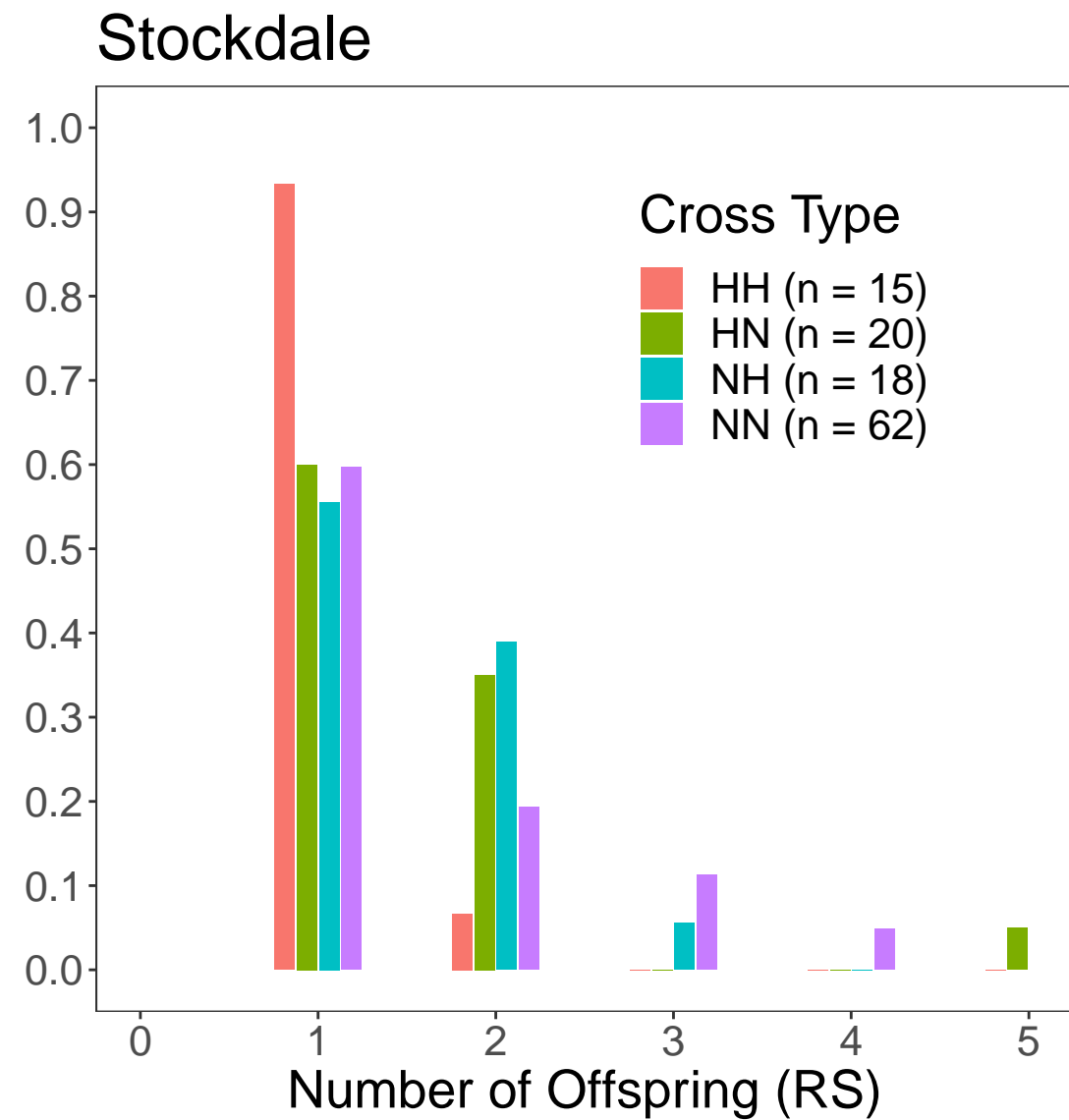

Supplement: Supplementary file 4 — Figure S4 [file EVA-15-429-s005.pdf]
