# Supplementary material for: Reduced relative fitness in hatchery‐origin Pink Salmon in two streams in Prince William Sound, Alaska
Source: Evol Appl. 2022 Mar 15;15(3):429–46. doi: 10.1111/eva.13356 (PMC8965367; doi:10.1111/eva.13356)
Supplement: Supplementary file 6 — Table S2‐S9 [file EVA-15-429-s006.docx]

# Supplemental Tables

Table S1. Summary of amplicons used to determine parentage of pink salmon samples including amplicon name, sequence, forward and reverse primer sequence, starting base pair and length, positions of SNPs within amplicon, and total length.

See attached .csv file

Table S2. Final sample sizes of Pink Salmon from Hogan Bay (Hogan) and Stockdale Creek (Stockdale) and samples removed during quality assurance for each brood year. “Missing” refers to individuals missing genotypes at more than 20% of loci. “Duplicate” indicates individuals removed because they share genotypes at 95% or greater of loci, likely due to the same tissues being sampled or DNA extracted twice. “Heterozygosity” refers to individuals removed because of outlying measures of heterozygosity, determined by 1.5 interquartile range cut-offs, suggesting contamination among individuals. “Final n” indicates the number of individuals retained and used in parentage analysis. “Percent retained” is the final n divided by the number initially genotyped.

| Stream | Year | Origin | Genotyped | Missing | Duplicate | Heterozygosity | Final n | % Retained |
| --- | --- | --- | --- | --- | --- | --- | --- | --- |
| Hogan | 2013 | Natural | 334 | 12 | 0 | 1 | 321 | 99 |
|  | 2013 | Hatchery | 461 | 12 | 4 | 3 | 442 | 96 |
|  | 2014 | Natural | 239 | 19 | 0 | 6 | 214 | 90 |
|  | 2014 | Hatchery | 512 | 51 | 2 | 22 | 437 | 85 |
|  | 2015 | Natural | 4,166 | 101 | 4 | 286 | 3,775 | 91 |
|  | 2016 | Natural | 4,295 | 117 | 12 | 172 | 3,994 | 93 |
|  | Total |  | 10,007 | 312 | 22 | 490 | 9,183 | 92 |
| Stockdale | 2013 | Natural | 871 | 54 | 0 | 6 | 811 | 93 |
|  | 2013 | Hatchery | 182 | 19 | 0 | 0 | 163 | 90 |
|  | 2014 | Natural | 436 | 65 | 4 | 9 | 358 | 82 |
|  | 2014 | Hatchery | 512 | 66 | 0 | 10 | 436 | 85 |
|  | 2015 | Natural | 6,667 | 355 | 20 | 239 | 6,053 | 91 |
|  | 2016 | Natural | 7,038 | 1,560 | 46 | 233 | 5,199 | 74 |
|  | Total |  | 15,706 | 2,119 | 70 | 497 | 13,020 | 83 |

Table S3. Final numbers of individual Pink Salmon from Hogan Bay and Stockdale Creek that were genotyped (after removing individuals during quality control and quality assurance (Table S1)), estimated escapements for each brood year, and estimated percent of escapements genotyped (% of Escapement), within each lineage (odd and even). Escapement estimates (in thousands) come from area under the curve (AUC) expansions of aerial survey and foot survey data (Steve Moffit, unpublished data).

| Hogan Bay Odd (2013/2015) | | | | |
| --- | --- | --- | --- | --- |
|  | Year | Total | Escapement | % of Escapement |
| Parents | 2013 | 763 | 8-47K | 2-10% |
| Offspring | 2015 | 3,775 | 10-19K | 20-38% |
| Total |  | 4,538 |  |  |
|  |  |  |  |  |
| Hogan Bay Even (2014/2016) | | | | |
|  | Year | Total | Escapement | % of Escapement |
| Parents | 2014 | 651 | 6-9K | 8-13% |
| Offspring | 2016 | 3,994 | 8K | 54% |
| Total |  | 4,645 |  |  |
|  |  |  |  |  |
| Stockdale Creek Odd (2013/2015) | | | | |
|  | Year | Total | Escapement | % of Escapement |
| Parents | 2013 | 974 | 4-11K | 9-24% |
| Offspring | 2015 | 6,053 | 25-27K | 22-24% |
| Total |  | 7,027 |  |  |
|  |  |  |  |  |
| Stockdale Creek Even (2014/2016) | | | | |
|  | Year | Total | Escapement | % of Escapement |
| Parents | 2014 | 794 | 2-4K | 20-40% |
| Offspring | 2016 | 5,199 | 26K | 20% |
| Total |  | 5,993 |  |  |

Table S4. Number of Pink Salmon sampled from Hogan Bay (Hogan) and Stockdale Creek (Stockdale) in each year, numbers of otoliths read and assigned as hatchery-origin (AFK = Armin F. Koernig, CC = Cannery Creek, SG = Solomon Gulch, WN = Wally Noerenberg) or identified as natural-origin. Percent hatchery-origin fish (pHOS) represents the weighted percent of hatchery fish in the escapement (Gorman et al., 2018; Knudsen et al., 2021). See Figure 1 for hatchery and stream locations.

| Stream | Year | Fish | Otoliths | Hatchery | | | | | Hatchery | Natural | pHOS |
| --- | --- | --- | --- | --- | --- | --- | --- | --- | --- | --- | --- |
|  |  | Collected | Read | AFK | CC | WN | SG | N/A | Total | Total |  |
| Hogan | 2013 | 829 | 799 | 366 | 13 | 86 | 0 | 0 | 465 | 334 | 64% |
|  | 2014 | 2,651 | 2,572 | 1,688 | 217 | 425 | 2 | 0 | 2,332 | 240 | 92% |
|  | 2015 | 9,441 | 9,033 | 2,624 | 1,108 | 794 | 325 | 2 | 4,853 | 4,180 | 58% |
|  | 2016 | 13,007 | 12,830 | 2,090 | 267 | 164 | 80 | 0 | 2,601 | 10,229 | 21% |
|  | Total | 25,928 | 25,234 | 6,768 | 1,605 | 1,469 | 407 | 2 | 10,251 | 14,983 |  |
| Stockdale | 2013 | 1,200 | 1,172 | 183 | 1 | 2 | 0 | 0 | 186 | 986 | 16% |
|  | 2014 | 1,551 | 1,518 | 1,039 | 28 | 2 | 0 | 0 | 1,069 | 449 | 74% |
|  | 2015 | 8,602 | 8,368 | 1,203 | 305 | 79 | 75 | 0 | 1,662 | 6,706 | 24% |
|  | 2016 | 9,000 | 8,749 | 624 | 5 | 3 | 7 | 0 | 639 | 8,110 | 8% |
|  | Total | 20,353 | 19,807 | 3,049 | 339 | 86 | 82 | 0 | 3,556 | 16,251 |  |

Table S5. Parameters used for *FRANz* (Riester et al., 2009) simulations (runs 1) and for parentage analysis (runs 1, 2, and 3) of Pink Salmon from Hogan Bay (Hogan) and Stockdale Creek (Stockdale) for odd- and even-year lineages. Escapement estimates are reported in (Stopha, 2016, 2017; Vercessi, 2014; Vercessi, 2015) and the genotyping error rate reflects the project’s overall error rate (0.5%). N_m_ – and N_fmax_ define the maximum number of potential parents (males and females). We used N_m_ – and N_fmax_ values that were approximately half of the estimates of escapement derived from aerial surveys and stream walks and assumed that approximately half of the fish were male and half were female. For Stockdale Odd runs 2 and 3 and Even run 2, we used fractions of escapement estimates. Genotyping error rates were derived from quality control and calculated following (Dann, et al. 2012). For run 3 in each stream and lineage we used an inflated genotyping error rate (1.0%) to determine the robustness of parentage assignments.

| Stream | Lineage | Run | N_m_- and N_fmax_ | Escapement Estimate | Genotyping Error Rate (%) |
| --- | --- | --- | --- | --- | --- |
| Hogan | Odd | 1 | 23,500 | Aerial survey | 0.5 |
|  |  | 2 | 4,300 | Stream walk | 0.5 |
|  |  | 3 | 23,500 | Aerial survey | 1.0 |
|  | Even | 1 | 4,500 | Aerial survey | 0.5 |
|  |  | 2 | 3,300 | Stream walk | 0.5 |
|  |  | 3 | 4,500 | Aerial survey | 1.0 |
| Stockdale | Odd | 1 | 4,150 | Aerial survey | 0.6 |
|  |  | 2 | 5,659 | ½ stream walk | 0.6 |
|  |  | 3 | 5,659 | ½ stream walk | 1.0 |
|  | Even | 1 | 4,150 | Stream walk | 0.6 |
|  |  | 2 | 2,019 | 1/10 of Aerial survey | 0.6 |
|  |  | 3 | 20,190 | Aerial survey | 1.0 |

Table S6. Number of families identified and mean unweighted reproductive success (RS) for each cross type for Hogan Bay (Hogan) and Stockdale Creek (Stockdale) even-year lineage (2014). NN = two natural-origin fish; NH = natural-origin female and hatchery-origin male; HN = hatchery-origin female and natural-origin male; HH = two hatchery-origin fish. Relative reproductive success (RRS) is the RS of each cross type divided by the RS of NN matings. Confidence intervals were calculated following (Kalinowski & Taper, 2005).

| Stream | Cross Type | Number of Families | Mean RS | RRS (95% confidence intervals) |
| --- | --- | --- | --- | --- |
| Hogan | NN | 4 | 1.25 | 1.00 |
|  | NH | 5 | 1.60 | 1.33 (0.40-4.79) |
|  | HN | 2 | 2.00 | 1.60 (0.40-5.00) |
|  | HH | 3 | 1.67 | 1.28 (0.43-4.24) |
| Stockdale | NN | 62 | 1.76 | 1.00 |
|  | NH | 18 | 1.50 | 0.85 (0.55-1.28) |
|  | HN | 20 | 1.55 | 0.88 (0.58-1.30) |
|  | HH | 15 | 1.07 | 0.61 (0.35-0.99) |

Table S7. Correlations among explanatory variables included in generalized linear models for Pink Salmon from Hogan Bay (Hogan) and Stockdale Creek (Stockdale) in 2014. These correlations were calculated by pooling all assigned parents (males and females) together.

| Stream | Explanatory Variables | Parent Length (mm) | Parent Sample Location (m) | Reproductive Success |
| --- | --- | --- | --- | --- |
| Hogan | Parent Sample Date | 0.28 | -0.10 | 0.03 |
|  | Parent Length (mm) |  | 0.12 | 0.07 |
|  | Parent Sample Location (m) |  |  | -0.10 |
| Stockdale | Parent Sample Date | 0.26 | 0.22 | -0.25 |
|  | Parent Length (mm) |  | 0.21 | 0.05 |
|  | Parent Sample Location (m) |  |  | -0.37 |

Table S8. Top 10 generalized linear models for parents from Hogan Bay collected in 2014, determined using Akaike’s Information Criterion (AIC), associating reproductive success with explanatory variables for females and males. DF = degrees of freedom.

| Female models | % Deviance Explained | AIC Weight | DF |
| --- | --- | --- | --- |
| Date + Length + Origin + Intertidal | 6% | 0.25 | 6 |
| Date + Length + Origin | 5% | 0.14 | 5 |
| Length + Origin | 4% | 0.14 | 4 |
| Date + Length + Origin + Distance | 5% | 0.10 | 6 |
| Intertidal + Origin | 5% | 0.07 | 4 |
| Length*Origin | 4% | 0.06 | 5 |
| Date*Origin | 6% | 0.05 | 5 |
| Date + Intertidal + Origin | 6% | 0.05 | 5 |
| Intertidal*Origin | 5% | 0.03 | 5 |
| Origin | 4% | 0.03 | 3 |
|  |  |  |  |
| Male models | % Deviance Explained | AIC Weight | DF |
| Length + Distance | 4% | 0.34 | 4 |
| Length | 2% | 0.13 | 3 |
| Date + Length + Distance | 4% | 0.13 | 5 |
| Length + Origin | 3% | 0.11 | 4 |
| Date + Length + Origin + Distance | 5% | 0.06 | 6 |
| Length + Intertidal | 3% | 0.06 | 4 |
| Length*Origin | 4% | 0.05 | 5 |
| Date + Length + Origin | 3% | 0.04 | 5 |
| Distance*Origin | 7% | 0.02 | 5 |
| Date + Length + Intertidal | 3% | 0.02 | 5 |

Table S9. Top 10 generalized linear models for parents from Stockdale Creek collected in 2014, determined using Akaike’s Information Criterion (AIC), associating reproductive success with explanatory variables for females and males. DF = degrees of freedom.

| Female models | % Deviance Explained | AIC Weight | DF |
| --- | --- | --- | --- |
| Date + Length + Origin + Distance | 25% | 0.99 | 6 |
| Date + Length + Distance | 22% | 0.01 | 5 |
| Length + Distance | 22% | 0.00 | 4 |
| Date + Length + Origin + Intertidal | 22% | 0.00 | 6 |
| Date + Origin | 9% | 0.00 | 4 |
| Date + Distance | 21% | 0.00 | 4 |
| Date + Distance + Origin | 23% | 0.00 | 5 |
| Distance + Origin | 23% | 0.00 | 4 |
| Distance*Origin | 23% | 0.00 | 5 |
| Date*Origin | 9% | 0.00 | 5 |
|  |  |  |  |
| Male models | % Deviance Explained | AIC Weight | DF |
| Date + Length + Origin + Distance | 36% | 1.00 | 6 |
| Date + Origin | 17% | 0.00 | 4 |
| Date + Distance | 22% | 0.00 | 4 |
| Date + Distance + Origin | 28% | 0.00 | 5 |
| Distance + Origin | 27% | 0.00 | 4 |
| Distance*Origin | 27% | 0.00 | 5 |
| Date*Origin | 18% | 0.00 | 5 |
| Length*Origin | 19% | 0.00 | 5 |
| Date + Length + Origin + Intertidal | 30% | 0.00 | 6 |
| Intertidal | 6% | 0.00 | 3 |

Figure S1. Schematic diagram showing genetic connections between hatchery (large ovals) and wild (small ovals) Pink Salmon in Prince William Sound. The odd- and even-year lineages are genetically distinct and non-overlapping. Within each lineage, hatcheries were founded with broodstock from local populations from 1976 to 1982 and use returning hatchery fish for broodstock in subsequent generations (i.e., segregated broodstock hatchery operation); changes in color over generations signify potential domestication over 15 to 20 generations. Streams have spawners of both natural- and hatchery-origin; changes in color over generations signify hatchery introgression. The full Alaska Hatchery Research Program will cover four years in each lineage for five streams; this paper covers the first two generations at two streams.

Figure S2. Distribution of individual genetic heterozygosity with mean and 1.5 inter-quartile range (IQR; red) and 3 standard deviations (SD; blue) cut-offs for Pink Salmon offspring and adults from the odd (2013/2015; top) and even (2014/2016; bottom) lineages in Hogan Bay and Stockdale Creek, Prince William Sound. Individuals outside of the IQR cut-offs were excluded from further analysis because they likely represented genetically contaminated samples.

Figure S3. Log likelihood ratios (logl_ratio) of true versus hypothesized relationships, calculated using CKMRSim (Anderson, DOI: 10.5281/zenodo.820162), implemented in R for Hogan and Stockdale, odd (2013) and even (2014). These distributions demonstrate that our SNP panel has the power to distinguish between true parent-offspring relationships and unrelated individuals. Since there is no ambiguity about the age of our fish, we can exclude full-siblings and half-siblings as potential results.

Figure S4. Distribution of family size by cross type for even-year lineage Pink Salmon parents collected in 2014 from Hogan Bay (Hogan) and Stockdale Creek (Stockdale), Prince William Sound. First letter in the cross designates the origin of the female (H = hatchery, N = natural) and second letter designates the origin of the male with n indicating the total number of families for each cross type. Note that only families with sampled offspring can be measured, so cross types for families producing zero sampled offspring cannot be represented. While there were few cross types for Hogan Bay, Stockdale Creek NN families tended to produce more offspring than HH families, with HN and NH families producing intermediate numbers of offspring on average.

# Supplemental Methods

## Otolith Analysis

Otoliths (left otolith from each pair) were mounted, sulcus side up, on petrographic glass slides with thermoplastic glue. Otoliths were wet-ground to the mid-sagittal plane at 250 rotations per minute using 500-grit SiC paper until the thermal mark or wild pattern (indicating hatchery- or natural-origin) could be seen through a compound light microscope at 200X magnification. Hatchery of origin was identified for otoliths with thermal marks based upon hatchery-specific banding patterns. If left otoliths were missing, fragmented, or over-ground, then right otoliths were read instead. All otolith readers passed randomized blind tests of known-origin fish to assess accuracy (Joyce & Evans, 1999). Approximately 30% of otolith trays were systematically selected to be read a second time by a different reader for quality control (QC). Any discrepancies between otolith reads were resolved by the supervisor. All reads (first, second, and supervisor overrides) were stored in a database and final reads were reported (Fernandez & Moffitt, 2016).

## Genotyping

### GT-seq modifications

We followed the Genotyping-in-Thousands by sequencing (GT-seq) methods described in (Campbell et al., 2015), other than deviations at the second PCR (PCR2), purification, and quantification steps as follows: 1) During PCR2, we used 2 µL of 10 µM well-specific i5 tag primers per well, bringing the final reaction volume to 11 µL. 2) During the purification step with magnetic beads, the final elution volume was increased to 17 µL and no additional TE pH 8.0 with 1% TWEEN 20 was added. 3) Quantitative PCR (qPCR) was completed using triplicate dilutions of 1:1000, 1:5000, and 1:10000. Four microliters of each dilution were used as template in 10 µL reactions using 6 µL Kapa Library Quantification Kit - Illumina/ROX Low (Kapa Biosystems, Wilmington, MA.) The qPCRs were performed in 384-well plates on a QuantStudio™ 12K Flex Real-Time PCR System (Life Technologies). Final dilutions of each plate library were normalized to 4 nM. The pooled library went through an additional purification step via magnetic beads, which involved adding 46.4 µL of Agencourt AMPure XP magnetic beads to 58 µL of pooled library. After incubation at room temperature for seven minutes, it was placed in a magnetic stand for five minutes and the supernatant was discarded. A double wash of 80% ethanol (ETOH) was performed for 30 seconds each. The tube incubated at room temperature for 5 minutes to dry off any residual ETOH. The elution was performed with 30 µL of 1X Low-EDTA TE, pH 8.0, incubated for five minutes before final transfer to a new 1.5 mL tube. The elution product was quantified for DNA yield via the manufacturer’s direction for the Qubit 3.0 (Thermo Fisher Scientific).

### Quality control

Laboratory quality control (QC) analysis was conducted by staff not involved in the original genotyping to identify laboratory errors and estimate the background error rate of the genotyping process (Dann et al., 2012). We re-extracted DNA from 8% of fish (approximately 2,866 fish total) and genotyped them for the same SNPs following the same methods. Human errors introduced during DNA extraction and genotyping identified in the QC process (e.g., accidental flipping of plates or rows that results in loss of pairing integrity between individual data and DNA) were resolved through additional extractions and genotyping and only corrected data were retained in the database. Discrepancy rates were calculated as the number of conflicting genotypes divided by the total number of genotypes compared. These rates describe the difference between project data and QC data for all SNPs. Discrepancy rates were divided by two to calculate the background error rate of original project genotyping, which assumes that genotyping errors are equally likely to occur during both genotyping processes and is reasonable given that both analyses use the same methods.

### Quality assurance

We imported genotypes into *R* (R Core Team, 2019) for additional quality assurance (QA) analyses using custom scripts (https://github.com/krshedd/GCL-R-Scripts). First, we removed individuals missing more than 20% of genotypes because they likely had poor-quality DNA and thus poor-quality genotypes. Second, we removed individuals sharing the same genotype in at least 95% of markers (i.e., duplicate genotypes, which can occur from sampling or extracting the same individual twice). Third, our QA analysis revealed that tissue degradation and/or contamination from multiple individuals resulted in individuals with excessively heterozygous multilocus genotypes that were not filtered out in the GTscore genotyping pipeline (Figure S2). We tested two heterozygosity filters: a +/- 3 standard deviation (SD) cutoff (Pettersson et al., 2011) and a cutoff of 1.5 interquartile range (IQR; Zar, 2010). We decided that the +/- 3 SD cutoff was not restrictive enough and inappropriate given our right-skewed distribution. We therefore chose to implement the 1.5 IQR cutoff to remove individuals with outlier heterozygosity values (Figure S2). This cutoff was applied separately to each lineage and stream.

# Supplemental Methods References

Campbell, N. R., Harmon, S. A., & Narum, S. R. (2015). Genotyping-in-Thousands by sequencing (GT-seq): A cost effective SNP genotyping method based on custom amplicon sequencing. *Molecular Ecology Resources*, *15*(4), 855–867. https://doi.org/10.1111/1755-0998.12357

Dann, T. H., Habicht, C., Jasper, J. R., Fox, E. K. C., Hoyt, H. A., Liller, H. L., Lardizabal, E. S., Kuriscak, P. A., Grauvogel, Z. D., & Templin, W. D. (2012). Sockeye salmon baseline for the Western Alaska salmon stock identification project. *Alaska Department of Fish and Game*, *Special Publication No. 12*-*12*, *Anchorage*. http://www.adfg.alaska.gov/FedAidpdfs/SP12-12.pdf

Fernandez, E., & Moffitt, S. D. (2016). Otolith processing and quality control methods used by the ADF&G Cordova Otolith Laboratory. *Alaska Hatchery Research Group*, *Technical Document 12*, 1–18. http://www.adfg.alaska.gov/static-f/fishing/PDFs/hatcheries/research/otolith_processing_methods_cordova.pdf

Joyce, T. L., & Evans, D. G. (1999). Otolith marking of pink salmon in Prince William Sound salmon hatcheries, Exon Valdez oil spill restoration final report (Restoration Project 99188). *Alaska Department of Fish and Game*, *Cordova*.

Pettersson, F. H., Anderson, C. A., Clarke, G. M., Barrett, J., Cardon, L. R., Morris, A. P., & Zondervan, K. T. (2009). Marker selection for genetic case-control association studies. *Nature Protocols*, *4*(5), 743–752. https://doi.org/10.1038/nprot.2009.38.Marker

R Core Team. (2019). *R: A language and environment for statistical computing*. R Foundation for Statistical Computing. https://www.r-project.org/

Zar, J. H. (2010). *Biostatistical Analysis* (5th ed.). Pearson.
